# Prevalent North Atlantic Deep Water during the Last Glacial Maximum and Heinrich Stadial 1

**DOI:** 10.1038/s41561-025-01685-5

**Published:** 2025-05-06

**Authors:** Patrick Blaser, Claire Waelbroeck, David J. R. Thornalley, Jörg Lippold, Frerk Pöppelmeier, Stefanie Kaboth-Bahr, Janne Repschläger, Samuel L. Jaccard

**Affiliations:** 1 Institute of Earth Sciences, University of Lausanne, Lausanne, Switzerland; 2 GEOMAR Helmholtz Centre for Ocean Research Kiel, Kiel, Germany; 3 LOCEAN/IPSL, Sorbonne Université-CNRS-IRD-MNHN, Paris, France; 4 Department of Geography, University College London, London, UK; 5 Institute of Earth Sciences, Heidelberg University, Heidelberg, Germany; 6 Climate and Environmental Physics, Physics Institute, University of Bern, Bern, Switzerland; 7 Oeschger Centre for Climate Change Research, University of Bern, Bern, Switzerland; 8 Institute of Geological Sciences, Freie Universität Berlin, Berlin, Germany; 9 Department of Climate Geochemistry, Max-Planck Institute for Chemistry, Mainz, Germany

**Keywords:** Palaeoceanography, Palaeoclimate

## Abstract

Deep ocean circulation modulated glacial–interglacial climates through feedbacks to the carbon cycle and energy distribution. Past work has suggested that contraction of well-ventilated North Atlantic Deep Water during glacial times facilitated carbon storage in the deep ocean and drawdown of atmospheric CO_2_ levels. However, the spatial extent and properties of different water masses remain uncertain, in part due to conflicting palaeoceanographic proxy reconstructions. Here we combine five independent proxies to increase confidence and reconstruct Atlantic deep water distributions during the Last Glacial Maximum (around 21 thousand years ago) and the following Heinrich Stadial 1—a time when massive ice rafting in the North Atlantic interfered with deep water formation and caused global climate shifts. We find that North Atlantic Deep Water remained widespread in both periods, although its properties shifted from a cold, well-ventilated mode to a less-ventilated, possibly warmer, mode. This finding implies a remarkable persistence of deep water formation under these cold boundary conditions, sustained by compensation between the two formation modes. Our constraints provide an important benchmark for evaluating Earth system models, which can enhance confidence in future climate projections.

## Abstract (web summary)

North Atlantic Deep Water formation was only moderately weaker than it is now during the last glacial period, even when freshwater inputs were high, according to an analysis of independent proxy records.

## Main

Ocean circulation plays a fundamental role in the climate system through its leverage on the global transport of heat, carbon and nutrients^1^. Reconstructions of past climates provide a unique opportunity to observe how the Earth system can respond to different forcings and boundary conditions. However, available palaeoceanographic reconstructions appear conflicting, even for relatively well-documented climate intervals such as the Last Glacial Maximum (LGM; 23–19 thousand years before present, ka bp) and the Heinrich Stadial 1 (HS1; 17.5–14.6 ka bp)^2^, limiting our ability to understand if and how ocean circulation changed during these periods.

Today’s Atlantic deep water geometry, which is described by properties such as temperature, salinity and nutrient concentrations^3^, reveals that North Atlantic Deep Water (NADW) fills most of the Atlantic Basin before feeding into the Antarctic Circumpolar Current^4,5^. By contrast, the abyssal plains of the South Atlantic are bathed by dense and less-ventilated Antarctic Bottom Water (AABW), which flows northwards and contributes up to 25% of the water mass mixture in the abyssal Northwest Atlantic^3^.

**Fig. 1:**
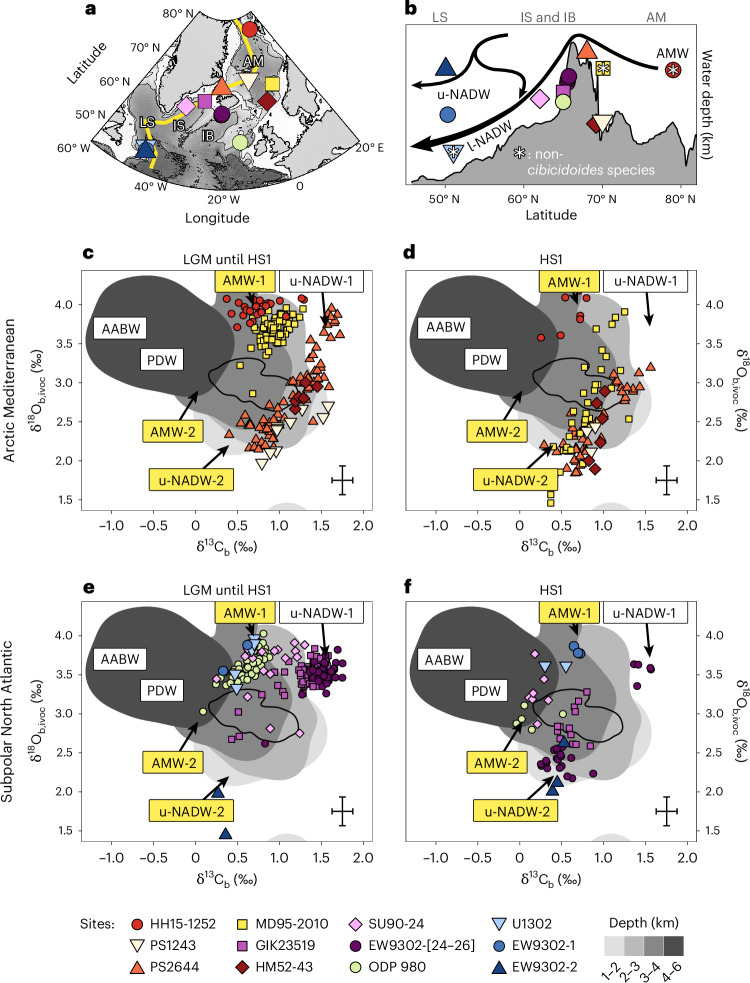
Benthic carbon and oxygen isotope data from across the subpolar North Atlantic and Arctic Mediterranean. **a**, Map showing the selected sediment core sites. These sites cover different regions of young NSW with less than 5% SSW today. AM, Arctic Mediterranean; LS, Labrador Sea; IS, Irminger Sea; IB, Iceland Basin. **b**, Section along the yellow line in **a** with the sediment sites shown. Arrows indicate modern NADW tributary water flows (see text). Data from non-*Cibicidoides* species (denoted by the asterisks) were corrected for species-specific offsets (Methods). **c**–**f**, Relationship between δ^13^C_b_ and δ^18^O_b,ivoc_ signatures^34,42,51,53,55^^,^^58–63^ for the LGM to HS1 (**c**,**e**) and HS1 (**d**,**f**) intervals (23–14.6 ka bp) for core sites in the Arctic Mediterranean (**c**,**d**) and subpolar North Atlantic (**e**,**f**). The background greyscale areas indicate compiled West Atlantic data according to their water depths. The black outline in each marks the full distribution of modern Atlantic seawater data from a >2 km water depth. Labels indicate the isotopic signatures of different glacial source waters, with those introduced in this study highlighted in yellow. All δ^18^O data are corrected for continental ice-volume (ivc) changes and where possible for core-top seawater offset (oc, and subscript ivoc for both corrections)—see Methods. The error cross indicates typical double standard deviation uncertainties including those from ivc. See Extended Data Fig. 1 for isotopic records. Basemap data in **a** from ref. ^64^.

**Fig. 2:**
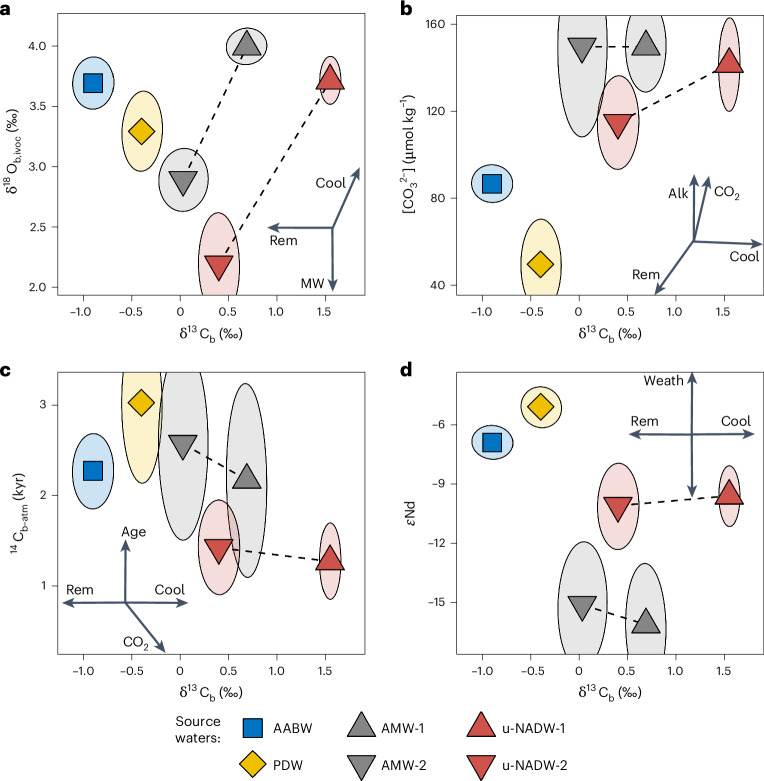
Atlantic source water signatures in different proxy–proxy spaces during the LGM. **a**–**d**, Relationship between δ^13^C_b_ and δ^18^O_b,ivoc_ (**a**), [CO_3_^2−^] (**b**), radiocarbon ventilation age ^14^C_b–atm_ (**c**) and εNd (**d**) signatures. Different source waters are shown as coloured symbols, and the estimated 95% level uncertainties are shown as error ellipses. Dashed lines connect source water modes 1 and 2 of the same northern source water type. Arrows on the inset axes indicate processes that can affect the proxy signatures of the source waters. Cool, surface water cooling; Rem, organic matter remineralization; MW, meteoric water admixture; Alk, alkalinity increase; CO_2_, CO_2_ evasion (slope depends on dynamics); Age, increase in carbon ventilation age; Weath, input of Nd through weathering. See Extended Data Fig. 2 for full source water proxy records and Extended Data Fig. 8 for source water signatures today.

NADW is today composed of two distinct source waters^6^: (1) upper NADW (u-NADW; alternatively termed Labrador Sea Water), which is produced in the subpolar North Atlantic (Fig. 1) and (2) lower NADW (l-NADW), formed by the overflow of dense water from the Arctic Mediterranean over the sills around Iceland into the deep North Atlantic and the entrainment of ambient subsurface water during its descent, which approximately doubles the initial volume flux^7–9^.

Palaeoceanographic reconstructions have long suggested that NADW was shallower and less vigorous during the LGM^10–13^. Although recent studies have challenged some of these findings^14–21^, the model still prevails that NADW was substantially less prevalent (that is, reduced in volume) during the LGM than during the Holocene. Consequently, the less-ventilated AABW and Pacific Deep Water^22^ (PDW) were more voluminous, which enabled enhanced sequestration of carbon from the atmosphere^23^.

Following the LGM, HS1 was marked by widespread iceberg rafting in the North Atlantic, leading to surface ocean freshening, which increased surface buoyancy and limited the potential for open-ocean deep convection^24,25^. It has been proposed that the Atlantic Meridional Overturning Circulation (AMOC) weakened substantially, curtailing meridional heat transport to the northern hemisphere^1^, and that the volumetric extent of NADW was reduced further. However, the associated deep water mass configuration and transport remain highly debated^26–33^.

The differences between apparently conflicting reconstructions of past deep water mixing are typically attributed to the uncertainties and biases of the proxies used, because they are influenced by processes beyond passive transport and conservative mixing with seawater, and the proxy signals can be modified by diagenesis. Here we integrate published data from five complementary proxies across the deep Atlantic in a consistent data-constrained framework to estimate the distribution of intermediate and deep water masses in the glacial Atlantic during the LGM and HS1 with increased confidence. We first identify the different potential source waters, and then determine how they may have combined to fill the deep Atlantic.

## Northern source waters during the late glacial period

**Extended Data Fig. 1:**
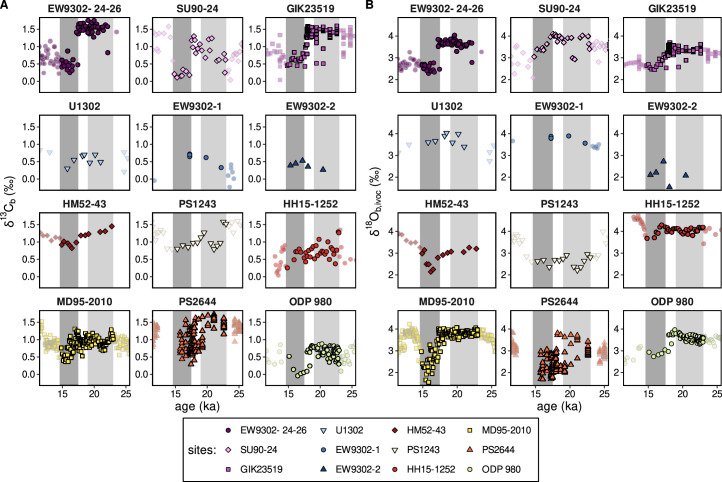
Carbon and oxygen isotope records from across the subpolar North Atlantic and Arctic Mediterranean. Sites are the same as in Fig. 1 and records are separated in carbon (**A**) and oxygen (**B**) isotopes. Dark and bright grey bars in background indicate timing of HS1 and LGM, respectively. Data outside of age range of Fig. 1 are shown brighter. See Supplementary Table 9 for data sources. Note that data from sites PS2644 and GIK23519 are analysed on individual benthic foraminifera, while the other records are based on averages of multiple specimens. See Fig. 1 and Supplement for data sources.

The compiled dataset supports earlier studies, indicating that u-NADW remained prevalent during the LGM^34–36^. The water mass was cold and generally well-ventilated, as indicated by elevated isotopic signatures of oxygen (δ^18^O_b_) and carbon (δ^13^C_b_) in the calcite shells of benthic foraminifera (Fig. 1 and Extended Data Fig. 1). These signatures were most pronounced during the LGM in the Iceland Basin at a water depth of between 1 and 2.5 km (ref. ^34^), whereas sites at greater depths and in the Arctic Mediterranean, the Labrador Sea and the Irminger Sea exhibited lower δ^13^C_b_ and, in part, lower δ^18^O_b_ as well. During HS1, the trend towards lower δ^18^O_b_ and δ^13^C_b_ became more pronounced and extended to the Iceland Basin at a depth of between 1 and 2.5 km.

These changes in proxy signatures at sites dominated by glacial u-NADW during HS1 have primarily been attributed to transformations in water mass characteristics rather than to increased admixture of southern-sourced water (SSW)^34,37^. This is also supported by neodymium isotope (εNd) proxy records, indicating a largely invariant water mass origin in the North Atlantic during the LGM and HS1^14,19,38–40^. From this, we propose that u-NADW prevailed during both the LGM and HS1, but experienced substantial changes in its stable carbon and oxygen isotopic signatures; we refer to these distinct modes of u-NADW, characterized by high and low isotopic ratios, as u-NADW-1 and u-NADW-2, respectively.

In addition to u-NADW-1 and u-NADW-2, a distinct cluster of data characterized by high δ^18^O_b_ and intermediate δ^13^C_b_ suggests the presence of an additional northern-sourced water (NSW) (Fig. 1). It was most prevalent at sites in the Arctic Mediterranean, and in the subpolar North Atlantic below 2 km depth. Accordingly, we identify this source water as glacial Arctic Mediterranean Water (AMW). The majority of sites characterized by AMW-like signatures show trends towards lower oxygen and carbon isotopic signatures during HS1. Given the similarity of these changes to those reported for the two u-NADW modes, we propose that AMW also existed in two distinct modes (AMW-1 and 2) with stable isotope signatures that varied similarly to those of u-NADW. Thus, we consider four modes of glacial NADW sources, varying in their contribution throughout the LGM and HS1: u-NADW-1, u-NADW-2, AMW-1 and AMW-2.

**Extended Data Fig. 2:**
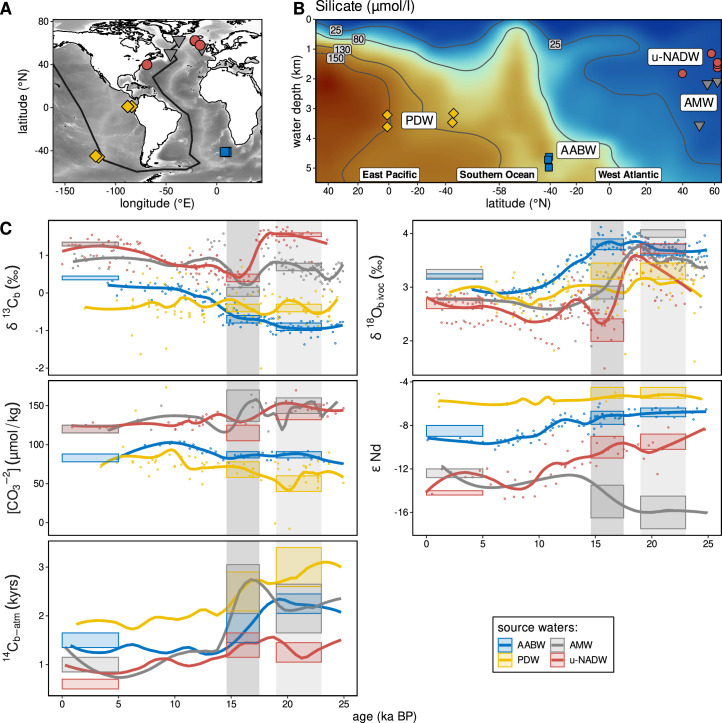
Source water proxy records. **a:** Map of sites assumed for source water definitions. **b:** Section along black line in A indicating the position of the source water sites with dissolved silicate concentration^94^ in background and labelled contours. **C:** Proxy records from source water sites^14,34,40,59,61,76,88,95–105^. Assumed source water signatures during LH, HS1, and LGM are marked as coloured boxes (height spanning 68% confidence interval). Vertical grey bars indicate time slices of HS1 (left) and LGM (right). See Methods and Supplement for details about source water end member sites and data sources used. Individual data points are shown together with LOESS trend lines. The trend for εNd of AMW is a smoothed estimate from ref. ^14^. Trend lines for radiocarbon ages are from ref. ^88^, based on binned and smoothed regional data compilations. Basemap data in **a** from ref. ^64^.

**Extended Data Fig. 3:**
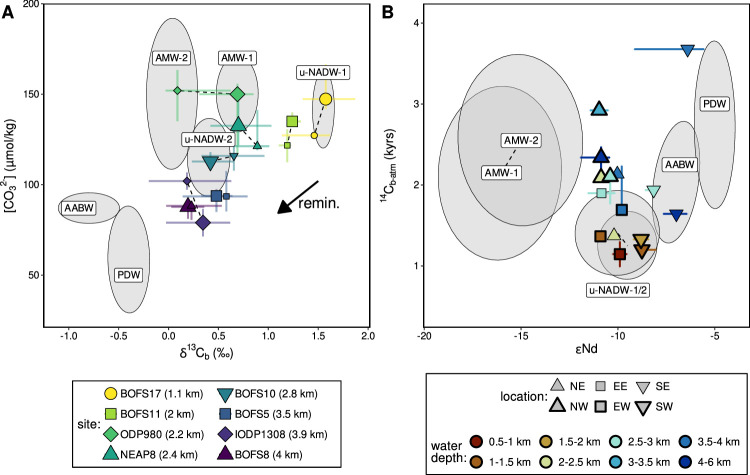
Additional figures for AMW proxy signature definitions. **a:** Relationship of carbonate ion concentration and carbon isotope signatures in the Iceland Basin. LGM and HS1 data for each site are shown as large and small symbols, respectively. Site depths are indicated in the legend. Site ODP 980 is located in the Rockall Trough, where Nordic Seas overflow waters are present today (Crocker et al.^38^). Black arrow indicates the relative changes the remineralisation of organic matter would bring about^35^, see also Fig. 2. **b:** relationship of εNd with radiocarbon age in Atlantic data from the LGM. Data are aggregated within different locations and depths because combined data from individual sites are barely available. Error bars and ellipses indicate 95% uncertainties or data spread.

The presence of glacial overflow waters (AMW) has often been overlooked, presumably because AMW was characterized by δ^13^C_b_ values intermediate between those of u-NADW and SSW, and thus it can be (mis)interpreted as a result of water mass mixing or enhanced organic matter remineralization. However, both δ^18^O_b_ and reconstructed carbonate ion concentration ([CO_3_^2−^]) values (Extended Data Figs. 2 and 3) strongly support the association of these intermediate δ^13^C_b_ signatures with a distinct source water originating in the Arctic Mediterranean^31,35,41,42^.

We note that not only data from HS1 but also some data from well-dated LGM sections trend towards lower δ^18^O_b_ and δ^13^C_b_, consistent with the existence of mode-2 waters (Fig. 1c,e). Thus, we suggest that the two modes (1 and 2) of both source waters (u-NADW and AMW) prevailed during both the LGM and HS1, but their relative contribution to the Atlantic water mass mixture varied strongly through time.

## Glacial NADW abundance estimated from multiple proxies

**Extended Data Fig. 4:**
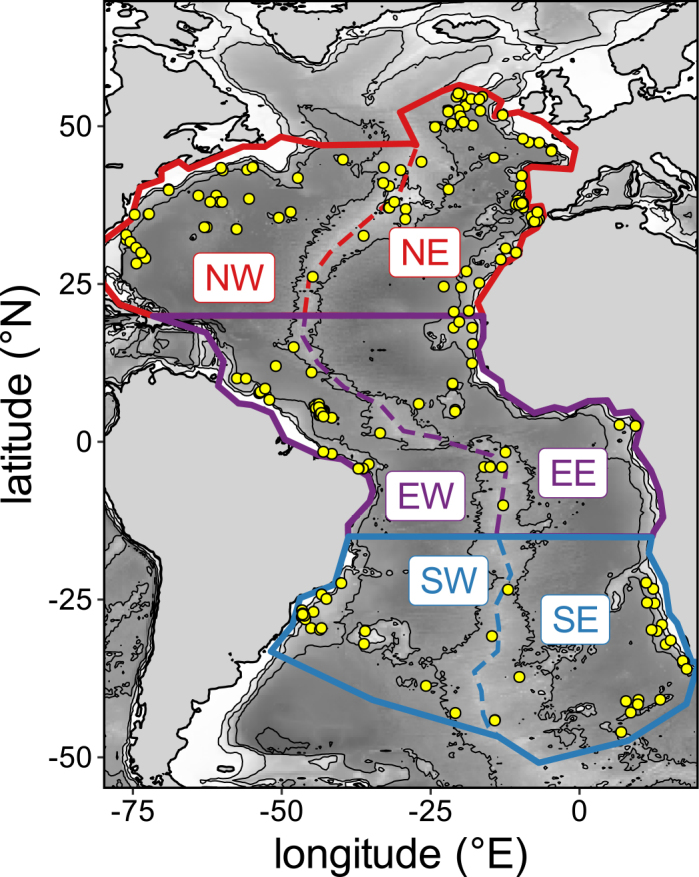
Spatial distribution of proxy data for the LGM and HS1. Yellow dots indicate positions of sediment cores contributing proxy observations. Different model regions are outlined and labelled. The distinction between Eastern and Western basins is only used for the box-pooled mixing calculations (see Extended Data Fig. 7). See Supplement for proxy data, locations, and references. Basemap data from ref. ^64^.

**Extended Data Fig. 5:**
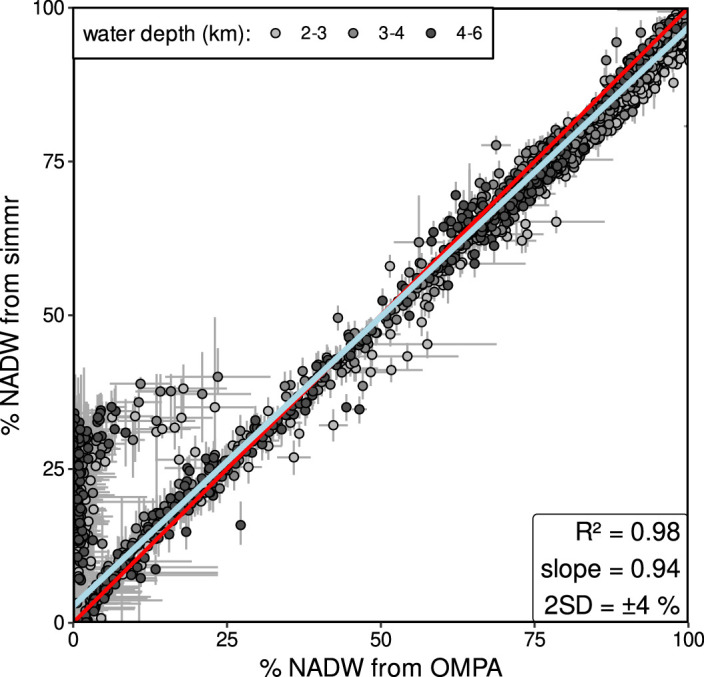
Modern source water identification test with the isotope mixing model used in this study (simmr). In this test, the simmr mixing model using physical and nutrient tracers (corrected for remineralisation via the Redfield ratio) is compared to published calculations via optimised multi parameter analyses (OMPA)^3^. Bright blue line shows linear regression and red line shows 1 to 1 correlation. Adjusted R-squared, the slope of the regression line and the prediction interval as double standard deviation are given in the box at the bottom right. Error bars indicate 95 % uncertainties. The regression was weighted by the inverse of the sum of both squared uncertainties.

**Extended Data Fig. 6:**
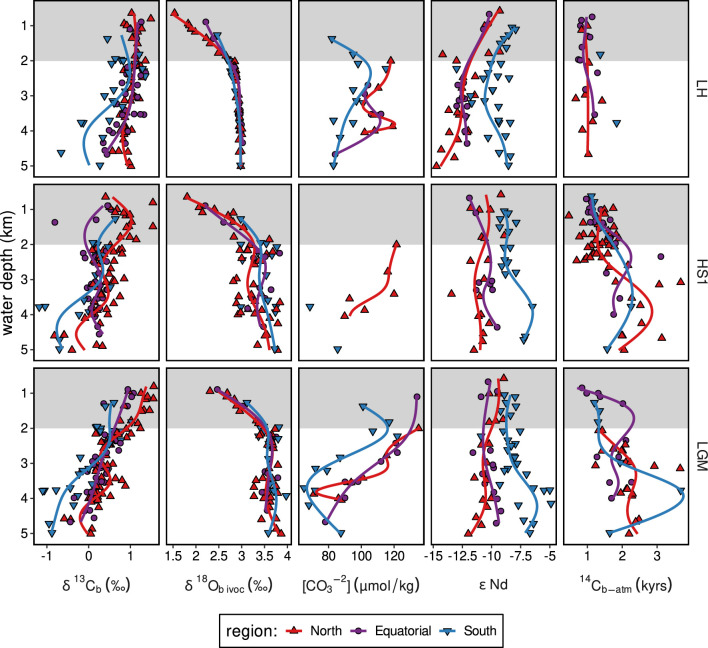
Depth-distributions of Atlantic proxy observations. The proxy data are split into three latitudinal boxes, each comprising both east and west here (see Extended Data Fig. 4). The lines are of proxy values smoothed and interpolated over depth with generalised additive models. δ^18^O_b_ data are corrected for both contemporary sea level effect and core top offsets from seawater estimates (ivoc). Uncertainties are not shown for simplicity.

**Extended Data Fig. 7:**
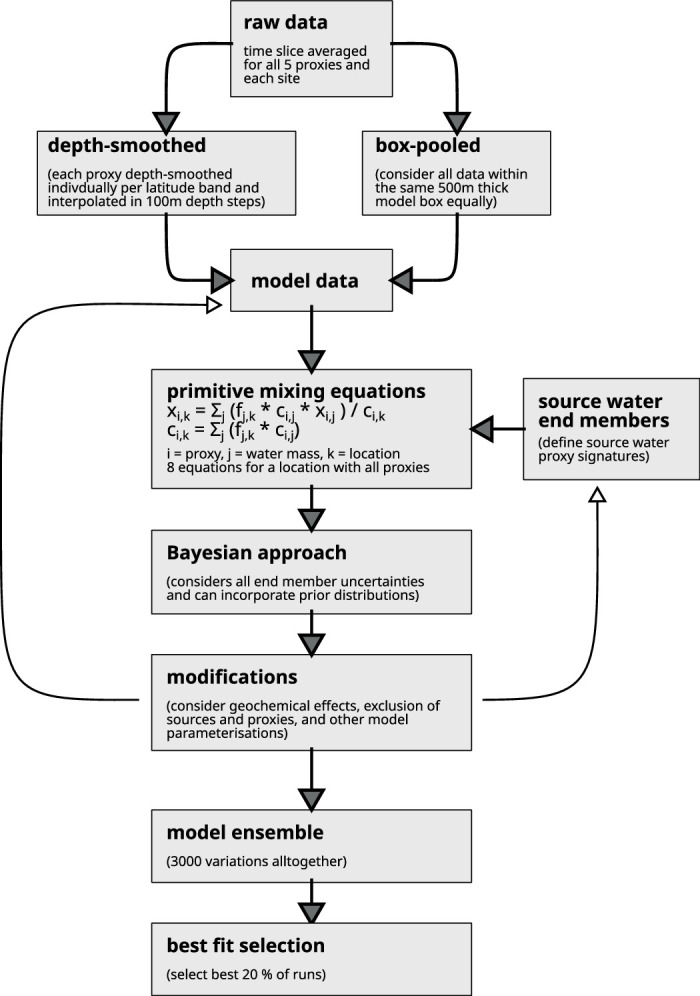
Scheme of the Bayesian mixing model. We used the model with two different data set aggregations: (**1**) depth-smoothed and interpolated data for the high resolution visualisation of source water layers in the North and South Atlantic (see main text) and (**2**) pooled data within 500 m thick model boxes for a more objective verification. The depth-smoothed approach is used for all discussions, numbers, and figures unless otherwise stated, and both approaches are compared in Extended Data Fig. 9. The model simply estimates mixing ratios that agree best with the proxy observations considering the uncertainties in the source water proxy signatures. Modifications of the relevant source water proxy signatures and the input data allows for simple forward parametrisations of geochemical effects on the proxy behaviours. We create a model ensemble comprising different realisations of such modifications and select the best fitting 20 % of realisations (see Supplementary Figs. 4 and 5) for evaluation in the main text and Figs. 3 and 4.

On the basis of the four newly defined components of NADW, we examine the composition of deep Atlantic water during the LGM, HS1 and late Holocene (LH). We consider water depths greater than 2 km, divide the Atlantic into six different regions and use a compilation of five geochemical proxies (that is, δ^13^C_b,_ δ^18^O_b,ivoc_, εNd, [CO_3_^2−^] and radiocarbon (^14^C) age reconstructions of deep waters—see Supplementary Text 1 and Extended Data Figs. 2 and 4). Considering reconstructions from the deep water source regions, we determine the proxy signatures for two SSWs (AABW and PDW) and the four contributors of NADW (u-NADW-1, u-NADW-2, AMW-1 and AMW-2)—see Fig. 2, Extended Data Figs. 2 and 3, Supplementary Text 2 and Supplementary Tables 4 and 5. These definitions of source water signatures enable us to quantify the relative contributions of the source waters using a Bayesian mixing model^43^ and the compiled proxy data (Methods and Extended Data Fig. 5). In this context, we first smooth the proxy observations with generalized additive models^44^ for three latitudinal Atlantic bands (north, equatorial and south; Extended Data Figs. 4 and 6). The simple mixing model enables us to efficiently estimate source water contributions that best fit all proxy observations simultaneously within each spatial box for the LGM, HS1 and LH. To test and account for geochemical processes affecting the observed proxies, we construct a large ensemble of 3,000 realizations, test the model set-up and apply a wide range of simple parametrizations of potentially important geochemical effects, such as the decomposition of organic matter for δ^13^C_b_ and [CO_3_^2−^] (ref. ^18^) or the exchange of Nd between bottom water and underlying sediments^45^ (Extended Data Fig. 7 and Methods).

**Fig. 3:**
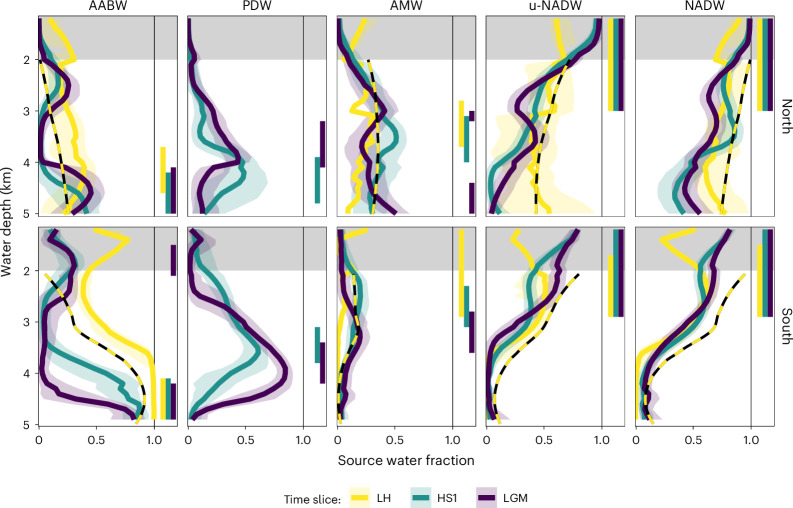
Source water distributions estimated from depth-smoothed proxy data for the North and South Atlantic. AMW, u-NADW and NADW comprise their two source water modes 1 and 2. Solid lines and shaded areas denote the averages and central 68% ranges, respectively, of the best fitting models for each time slice. Black–yellow dashed lines indicate modern distributions, where AMW and u-NADW were corrected for entrainment in l-NADW (Methods). Vertical bars at the right panel edges indicate the main depths of the source waters (running mean contributions of >0.75 of the maximum). Estimates for depths of <2 km (grey-shaded areas) are only shown for better visualization and are not quantitative due to the presence of Antarctic Intermediate Water, which is not included in the calculations. See also Extended Data Figs. 6 and 9. (NADW = u-NADW + AMW).

This analysis reveals that the glacial Atlantic source waters generally occupied a depth range similar to that observed today (Fig. 3), yet with two important differences. First, there was a shallower distribution of AMW (and hence l-NADW)—mostly restricted to depths above 3 km—in general agreement with previous studies^12,18^. Second, building on recent proxy-based studies^22,46^, our analysis indicates the replacement of AABW by the nutrient-rich PDW, that is, a source water characterized by very low [CO_3_^2−^] and high εNd (Extended Data Fig. 2), which penetrated northwards into the North Atlantic. We estimate that PDW contributed 28 ± 11% (median and 95% range) during both the LGM and HS1. It is noteworthy that this feature has not yet been reproduced by ocean circulation models despite emerging proxy evidence^22,46^.

**Extended Data Fig. 8:**
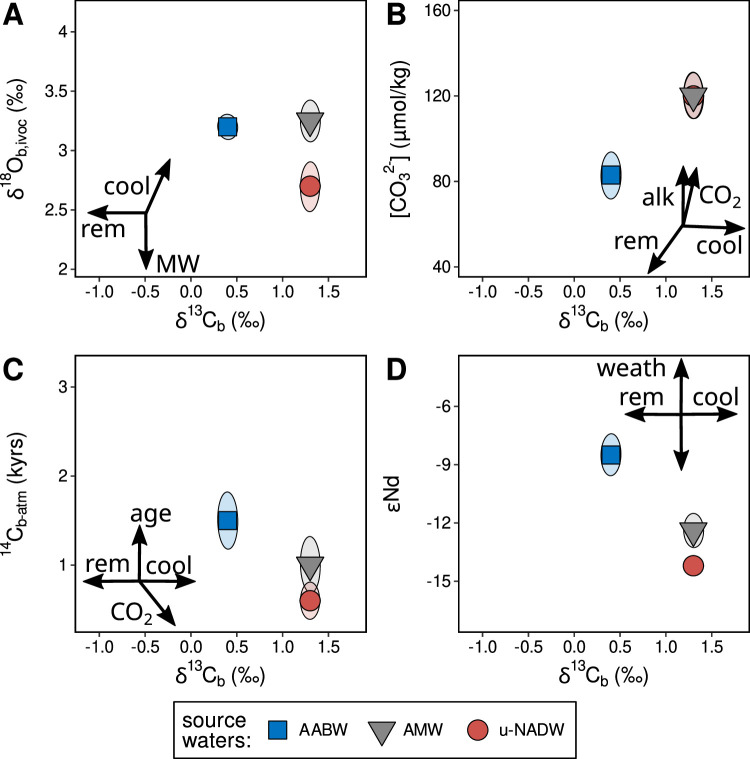
Atlantic source water signatures in different proxy-proxy spaces for modern and Late Holocene source waters. **A**–**D**, Relationship between δ^13^C_b_ and δ^18^O_b,ivoc_ (**A**), [CO_3_^2^^−^] (**B**), radiocarbon ventilation age ^14^C_b__–atm_ (**C**) and εNd (**D**) signatures. Panels, arrows and axis ranges are as in Fig. 2.

**Extended Data Fig. 9:**
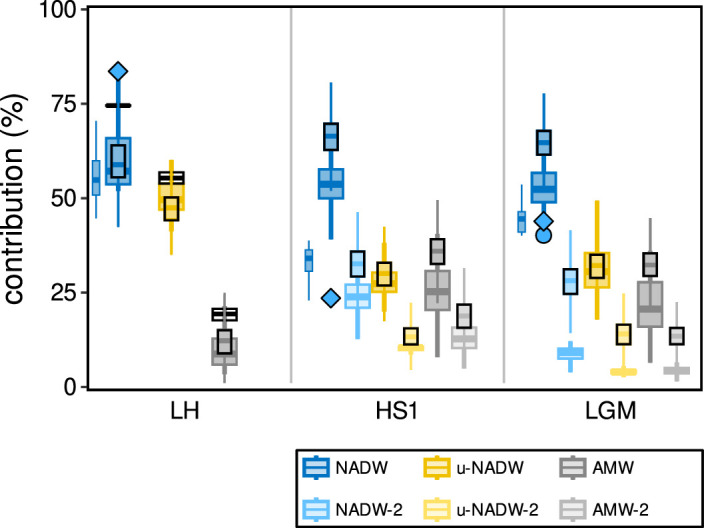
Best fitting mixing model ensemble results for average NADW contributions. Results of best fitting model runs (see Methods) are shown as boxes spanning 50% of data and including median lines and whiskers spanning 95% data ranges. Colours indicate different NADW types and modes (NADW-2 = u-NADW-2 + AMW-2; u-NADW = u-NADW-1 + u-NADW-2; AMW = AMW-1 + AMW-2). The thin blue NADW boxes are results from sub-ensembles without the suggested new source waters (that is NADW:= u-NADW-1). Black horizontal markers at Late Holocene (LH) are mean values estimated from analyses of oceanographic tracers in modern seawater. Blue diamonds and circle are results from refs. ^13,18^. All data are from the Atlantic below 2 km water depth. For the LH, contributions of u-NADW and l-NADW were estimated, and were converted to u-NADW and AMW by assuming 55 ± 5 % fractional entrainment of u-NADW in l-NADW for better comparison^3,8,13,18^. Overlain boxes with black borders are results from the box-pooled model ensemble as comparison.

Our estimated contribution of the combined NADW constituents in the deep Atlantic is remarkably invariant across the investigated time periods (Fig. 3). For the 20% best fitting model realizations, all of which consider all source waters, NADW contributions average to 52 ± 10% for the LGM, 53 ± 10% for HS1 and 57 ± 13% for the LH, compared with about 75% today as estimated on the basis of oceanographic tracers^3^. The fact that our estimate for the LH is significantly lower than those from oceanographic tracers may indicate a general bias in our proxy-based estimates, and that the real values for the LGM and HS1 may accordingly be somewhat higher than the estimates provided here. However, the LH reconstructions also suffer from the fact that source water proxy signatures span only small ranges, which may impact these analyses (Fig. 2 and Extended Data Fig. 8). Nonetheless, the contributions of glacial NADW are significantly higher than recent estimates of 42% and 24% for the LGM and HS1, respectively^13,18^. Better agreement with these values is achieved if we restrict NADW to its classical u-NADW-1 source water type, which reduces the median contribution of NADW estimated for the LGM and HS1 to 45 ± 9% and 34 ± 10%, respectively (Extended Data Fig. 9). This systematic bias underlines the importance of considering all NADW source waters, including both u-NADW and l-NADW or its precursor AMW, and their variable formation modes. Notably, the volumes of both u-NADW and AMW did not change significantly, but remained important during the LGM and HS1 (Fig. 3 and Extended Data Fig. 9).

**Fig. 4:**
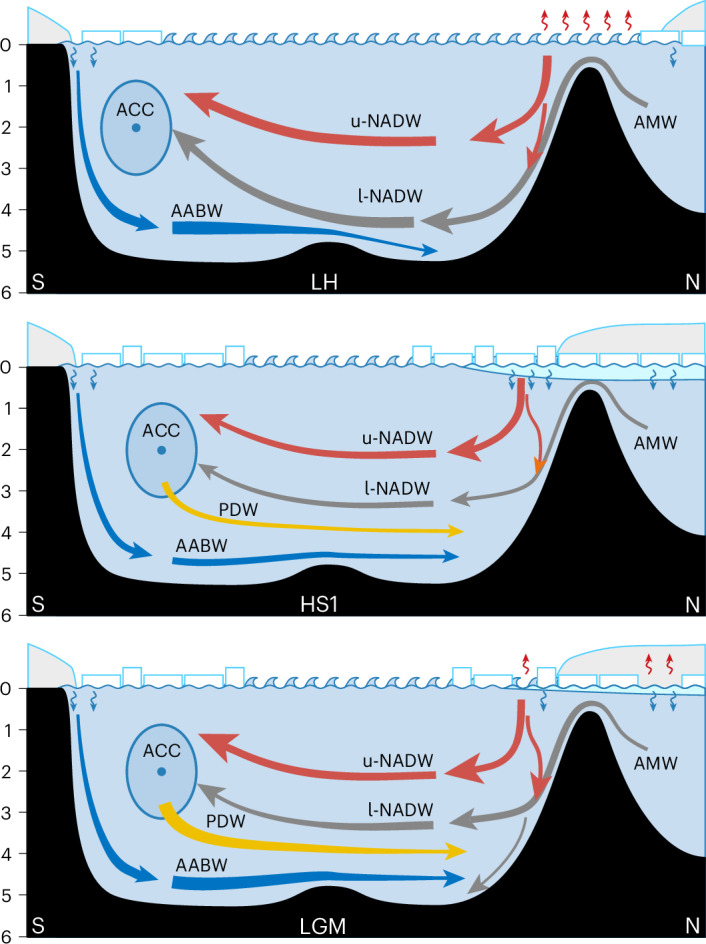
Schematics of possible AMOC during the three time slices LGM, HS1 and LH. Illustrations showing source water mixing in the Atlantic Basin during the LH (top), HS1 (middle) and LGM (bottom). Arrows indicate the major deep ocean water mass flows, with thickness qualitatively indicating the flux. Sea ice and icebergs are indicated as white rectangles; continental ice is represented by the light grey caps on top; freshwater is denoted by the cyan-blue layer below. Heat release is shown as small red arrows, and salt injection from sea-ice formation is shown as small blue arrows. Numbers along the left edge indicate the approximate water depth in kilometres. The most notable differences between the LGM and HS1 are the supposed weaker deep overturning and less heat release combined with more brine rejection in the North Atlantic during HS1. ACC, Antarctic Circumpolar Current; N, north; S, south.

**Extended Data Fig. 10:**
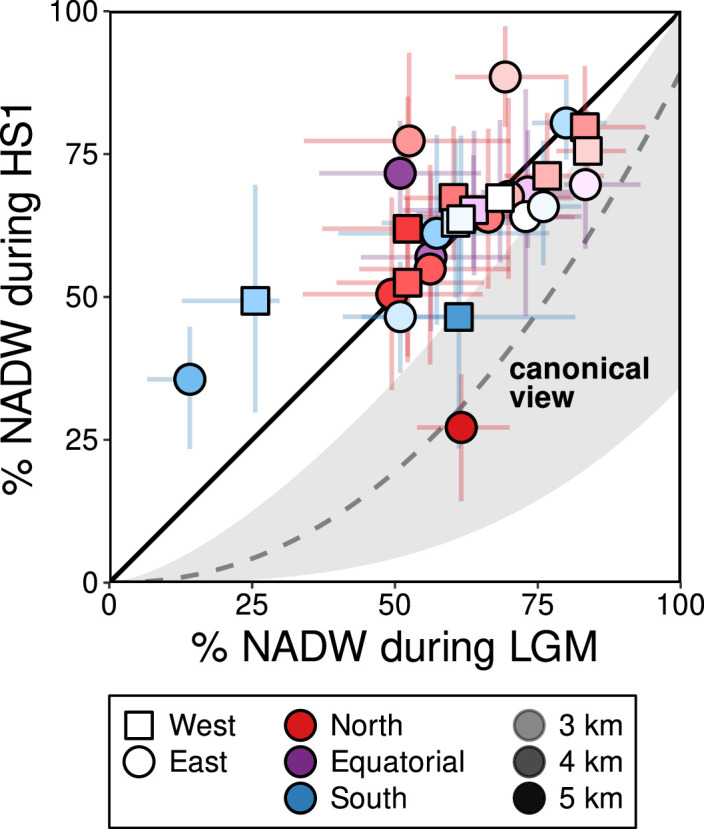
Comparison of mixing model ensemble results for NADW prevalence in the Atlantic during LGM and HS1. Results are from the box-pooled model approach and averaged across the 20 % best HS1 and LGM ensemble runs for each 500 m thick model box. Error bars indicate central 50 % of ensemble data. Black line shows 1:1 ratio. Symbol shapes distinguish West- from East Atlantic and fill colours indicate water depths. Grey area qualitatively indicates the field in which data are expected to lie if there had been a strong reduction of NADW prevalence during HS1.

For the LGM and HS1, both our estimated contribution of bulk NADW and its spatial distribution generally agree, and they are clearly at odds with a strong volumetric reduction of NADW in the Atlantic during HS1 (Extended Data Fig. 10). This observation is consistent with active deep water formation in the North Atlantic during HS1 (refs. ^28,31^), despite elevated freshwater input. The ratio of NSW to SSW volume in the Atlantic should be strongly influenced by both their relative densities and production rates, and hence the lack of a substantial reduction in NADW from the LGM to HS1 suggests that either relative densities and production rates remained similar or rather they changed in the same direction for both NSW and SSW. For example, a conceivable reduction in the production of both NADW and SSW during HS1 would reconcile our findings with independent evidence for a weakened AMOC during HS1 (refs. ^24,26^) (Fig. 4).

Although our analysis suggests that NADW volumetric contributions remained similar during the LGM and HS1, the proxy signatures (and hence the inferred water mass properties) changed. Our results show that these shifts can be attributed primarily to changes in the internal composition of NADW and SSW, and thus do not require major reorganizations of Atlantic source water provenance. In particular, proxy changes from LGM to HS1 appear to be caused by an increase of mode-2 NADW from 9 ± 3 to 24 ± 8% (Extended Data Fig. 9 and Supplementary Table 7).

## Mechanisms of glacial NADW formation

Open-ocean convection observed around the North Atlantic today is accompanied by ocean heat loss and air–sea gas exchange, and results in high δ^18^O_b_ and δ^13^C_b_ at depth. Whereas this is observed for glacial u-NADW-1, the δ^13^C_b_ signature of AMW-1 remained substantially lower (1.6 ± 0.1 versus 0.7 ± 0.1‰; Fig. 2). This is particularly important because the low δ^13^C_b_ of AMW is close to that of SSW, and hence additional proxies such as εNd or [CO_3_^2−^] are necessary for accurate water mass mixing estimates. The lower δ^13^C_b_ may have been caused by an increased imprint from organic matter remineralization from the Arctic Mediterranean^47^ or by suppressed air–sea gas exchange due to extensive sea-ice cover. This process has also been proposed as a cause for the particularly low δ^13^C_b_ of AABW in the Atlantic sector of the Southern Ocean^48,49^.

Our estimated proxy signatures of mode-2 source waters (u-NADW-2 and AMW-2) are characterized by lower δ^13^C_b_ and δ^18^O_b_ values than their mode-1 counterparts and generally similar εNd signatures^14,40^. There is also some less clear evidence for increased radiocarbon ventilation ages^50^ and slightly lower [CO_3_^2−^] for u-NADW-2 (ref. ^35^) (Fig. 2 and Extended Data Figs. 2 and 3). Whereas the similar εNd signatures between LGM and HS1 support invariant provenance of mode-1 and mode-2 waters, the disparities in the other proxies indicate considerable differences in their physical and chemical properties, presumably due to different formation processes.

Traditionally, low δ^18^O_b_ in the glacial North Atlantic and Arctic Mediterranean has been attributed to the incorporation of meteoric (melt)water with very low δ^18^O, transported to depth by dense brines produced during sea-ice formation^33,41,51,52^. Alternatively, higher temperatures could explain the low δ^18^O_b_ signatures of mode-2 source waters^53–55^. The required temperature differences between source water modes 1 and 2 would be large, with up to 4 and 6 °C for AMW and u-NADW, respectively. It appears unlikely that such warm but dense waters were formed by open-ocean convection. Instead, mixing with warm and saline subsurface water from the subtropical Atlantic or the Mediterranean Sea has been suggested to have led to warm product waters^28,31,56^. Yet, the low δ^13^C_b_ signatures combined with apparently higher radiocarbon ages in the mode-2 source waters still suggest that either surface gas exchange was reduced^48,57^ or they were mixed with a larger and older carbon pool, for example, from the Arctic Mediterranean.

## A revised view of water mass changes since the LGM

Our new analysis, based on five independent palaeoceanographic proxies, suggests that at least four distinct northern source deep waters co-existed in the Atlantic during the last ice age. Northern source deep water comprised a mixture of u-NADW and l-NADW like today, but in both well-ventilated (mode 1) and less-ventilated, potentially warmer (mode 2), variants. Contrary to proxy interpretations until now, our mixing calculations, which consider all source waters, show that, below a depth of 2 km, the volume of NADW in the Atlantic remained similar during the LGM and HS1, and only moderately lower than today. An increase in mode-2 deep water formation compensated for a decrease in mode-1 open-ocean convection around the North Atlantic during HS1, with associated changes in geochemical signatures and probably in air–sea gas and heat exchange. Potentially, HS1 deep water formation was weaker than during the LGM, but, to maintain the similar water mass distribution as reconstructed, this necessitates that other changes, such as reduced SSW production, compensated for any weakening of NADW production. Our new constraints provide an important target and testbed for assessing the ability of Earth system models to accurately simulate the response of Atlantic circulation to past climate forcing, ultimately providing increased confidence in their use for future projections.

## Methods

### Carbon and oxygen stable isotope data from the subpolar North Atlantic and Arctic Mediterranean

Sediment records with carbon and oxygen stable isotope data that span the LGM and HS1 were compiled from around the Arctic Mediterranean and the subpolar North Atlantic for Fig. 1 (Supplementary Table 10). Whereas most of these records are based on epifaunal benthic foraminifera of the *Cibicidoides* genus, we included three records that are based on infaunal foraminifera species, as indicated in Fig. 1 (sites U1302, MD95-2010 and HH15-1252PC). For infaunal foraminifera, the carbon and oxygen isotopic signatures do not directly reflect the dissolved inorganic carbon (DIC) of local bottom water and we thus applied constant interspecies corrections to account for species-specific fractionation in the oxygen and carbon isotopes. We assumed that isotopes in the shells of *Cibicidoides wuellerstorfi* are precipitated without biological fractionation and applied constant fractionation factors of −0.64‰ for δ^18^O_b_ (ref. ^65^) and +0.9‰ for the δ^13^C_b_ values^66^ of *Uvigerina peregrina* (site U1302)^67^. *Cassidulina neoteretis* data from site HH15-1252PC (ref. ^53^) and *Cassidulina teretis* data from site MD95-2010 (ref. ^51^) were assumed to be equally offset from equilibrium and were corrected by −0.64‰ for δ^18^O_b_ values^51^ and +1.5‰ for δ^13^C_b_. The correction for δ^13^C_b_ was obtained by alignment of data from site MD95-2010 and *Cibicidoides*-based data from site PS2644 across the available data overlap between 11.4 to 12.1 ka (20 data points of MD95-2010). This last correction certainly bears the largest potential for bias, but it is noteworthy that the overall interpretations related to Fig. 1 and the definition of the different components of NADW do not exclusively rely on data from non-*Cibicidoides* species and would not change significantly if only data from *Cibicidoides* records were considered.

Another potential problem is a low abundance of target foraminifera species such as *Cibicidoides*, which can promote biases through bioturbation moving these shells vertically in the sediment column. This has been suggested for the glacial section of site PS1243 (ref. ^47^). However, the data agree well with those of site HM52-43 and shallower site PS2644 (which has more abundant glacial *Cibicidoides*), potentially suggesting a well-preserved palaeoenvironmental signal.

### New carbon and oxygen stable isotope time-slice data

*Cibicidoides* δ^18^O_b_ and δ^13^C_b_ values from 19 sites across the Atlantic are derived from new records that were produced during the ACCLIMATE project and consistently dated by integrating radiocarbon ages and stratigraphic tie points using the Undatable software^2^ (age models can be downloaded from https://www.seanoe.org/data/00484/59554/). Epifaunal benthic foraminifers of the *Cibicidoides* genus were hand-picked in the >150 µm size fraction. The *C. wuellerstorfi* samples were picked when possible.

*Cibicidoides* oxygen and carbon isotope ratios for these samples were measured at the Laboratory for Climate and Environmental Sciences using a MicroMass IsoPrime100 mass spectrometer on samples of 1–5 specimens using the NBS-19 standard relative to the Vienna PeeDee Belemnite (VPDB). The mean external reproducibility of carbonate standards was 0.05‰ for δ^18^O and 0.03‰ (one standard deviation) for δ^13^C; the measured NBS-18 δ^18^O was −23.27 ± 0.10‰ VPDB, and δ^13^C was −5.01 ± 0.03‰ VPDB.

### Glacial Atlantic water mass proxy database

Proxy data for Atlantic deep water stable isotope signatures (δ^13^C_b_ and δ^18^O_b_), [CO_3_^2−^] values inferred from B/Ca ratios and radiocarbon ventilation ages (^14^C_b–atm_)—all measured on benthic foraminifera calcite—as well as radiogenic Nd isotope signatures (εNd), extracted from authigenic sediment phases via dissolution of foraminifera or acid-reductive bulk sediment leaching, were compiled from this study and several original publications and compilations^14–16,18,21,22,31,68–74^ (Extended Data Figs. 4 and 6 and Supplementary Table 13). For carbon and oxygen isotopes and B/Ca ratios, only foraminifera from the *Cibicidoides* genus, preferably *C. wuellerstorfi*, were used for this compilation. The Nd isotope data of five sites from the eastern subpolar North Atlantic were omitted because it has been suggested that they are compromised by localized non-conservative effects^75–77^. The data were averaged for each site for the LGM (23–19 ka bp), HS1 (17.5–14.6 ka bp) and LH (5–0 ka bp) on the basis of the existing age models from the same literature or updated age models within the ACCLIMATE project as described above. For completeness, we briefly describe each proxy in Supplementary Text 1. Carbon and oxygen stable isotope data from non-*Cibicidoides* foraminifera were not used for this compilation, but only for Fig. 1 as indicated in Fig. 1b.

### Ice-volume correction and core-top-offset correction of δ^18^O data

All δ^18^O_b_ values reported are corrected for global ice volume (δ^18^O_b,ivc_) by converting the contemporary sea level relative to modern to a related change in global marine δ^18^O. To this end, we used the sea-level curve from ref. ^78^ and assumed a sensitivity of 1.05‰ per 134.3 m sea-level change to enable a comparison across time periods (Extended Data Fig. 1 and Supplementary Figs. 1–3). The accuracy and consistency of this correction depends on the quality of the age models. This is particularly true for time intervals during which global sea level underwent drastic changes, such as the transition between Marine Isotope Stages 2 and 1. In the time intervals around the LGM and HS1, inaccuracies in the age models of, for example, 0.5 and 1.0 ka can lead to biases in the δ^18^O_b,ivc_ signals of up to 0.13 and 0.21‰, respectively (Supplementary Fig. 1). Whereas this source of uncertainty is comparable to measurement and offset correction uncertainties, it is still minor compared with the overall range of the δ^18^O_b,ivc_ that we estimate for the different glacial source waters (2.2–4.0‰).

It has been shown that analyses of oxygen isotope data from foraminifera may suffer from systematic biases due to gas mixing in the mass spectrometer source or other non-ideal instrument performance^31,79^. Assuming that such biases can be considered to be constant along a given δ^18^O_b_ record, we corrected the measured signals via a constant site-specific offset that minimizes the difference between the LH (here younger than 4 ka bp if available, otherwise <6 ka or <8 ka) data and the equilibrium *Cibicidoides* δ^18^O_b_ values computed from local seawater δ^18^O and temperature according to equation (9) from ref. ^54^. Local seawater δ^18^O, in turn, was inferred from local seawater salinity and basin- and depth-specific linear regressions of seawater δ^18^O versus salinity (Supplementary Table 1 and Supplementary Fig. 2). The regressions were generated from the seawater δ^18^O dataset of the Goddard Institute for Space Studies^80–82^. Local seawater temperature and salinity were interpolated from the World Ocean Atlas 2013 gridded global dataset^83,84^.

The offsets between LH foraminifera δ^18^O_b_ and equilibrium *Cibicidoides* δ^18^O_b_ average to a slightly positive value of 0.19 ± 0.56‰ (two standard deviations, *n* = 104). By adding these constant site-specific offsets, glacial δ^18^O_b,ivc_ values are therefore in average shifted towards slightly higher values and low outliers are reduced (Supplementary Fig. 3). The data thus appear more consistent. In particular, the data spread in water depths between 2 and 4 km is reduced. For example, uncorrected LGM δ^18^O_b,ivc_ data across the Atlantic below a 2 km water depth average to 3.27 ± 0.54‰, and offset-corrected data (δ^18^O_b,ivoc_) to 3.60 ± 0.49‰. Importantly, the reduced data spread tends to decrease the contribution of low-δ^18^O_b_ (mode 2) source waters in the mixing results.

### Multi-proxy mixing calculations

We estimated the relative contributions of different source waters in the deep Atlantic from the multi-proxy dataset using the ‘simmr’ package in the R programming language^43,85^ (Extended Data Fig. 7). The simmr package is a Bayesian stable isotope mixing model that uses Gibbs sampling and Markov chain Monte Carlo simulations, and it was originally developed for isotopic mixing calculations in ecological feeding studies but can be directly applied to other mixing scenarios. Starting from an a priori source probability distribution, simmr repeatedly samples the proxy space semi-randomly and tries to find mixing proportions of defined sources that agree with the observation(s). Proxy uncertainties of the sources are included, but not those of individual observations. Prior distributions can be used in the form of suggested source water probability distributions to improve the calculations with additional knowledge of the mixing system. Fixed proxy concentrations can be included and are here used in the form of DIC for δ^13^C and the ^14^C ventilation age and Nd concentrations for εNd in the different source waters. The method can cope with an arbitrary number of sources and proxies, but the larger the number of sources compared with proxies the more uncertain the results will be. A posteriori combination of sources can be used to reduce the uncertainty again, which we use, for example, for the estimation of bulk NADW and SSW. The simmr results are given as probability distributions from which we calculated the summary statistics. The choice of sources is critical (see below) and systematically affects the resulting mixing proportions (Supplementary Figs. 11, 14 and 15).

To estimate Atlantic-wide source water volumes we subdivided the Atlantic into three roughly latitudinal sections (Extended Data Fig. 4) and generated depth-smoothed profiles for each time slice and each proxy that was sufficiently available (Extended Data Fig. 6). We solve the simmr model for each latitudinal section and water depth independently, and the results between boxes are only linked via the intrinsic connection in the proxy data. Smoothing the proxy data emphasizes general trends in proxy and source water distributions. However, there is a limited amount of subjectivity related to the choice of smoothing parameters. Thus, we compare the results with an alternative and more objective approach, which we call box-pooling (Extended Data Fig. 7). For this approach, we subdivide the deep Atlantic into seven depth layers (from a water depth of 2 to 5 km in 0.5 km steps, and one box of >5 km) and increase the resolution by splitting each latitudinal section into east and west (Extended Data Fig. 4), resulting in 32 boxes containing observations during the LGM and HS1. This box-pooling approach is more objective because it does not include any other data treatment (such as smoothing or averaging across cores). Both approaches agree well overall (Extended Data Fig. 9), with the most obvious difference being that the box-pooling method leads to more mode-2 NADW in the LGM. We suggest that this is due to the proxy data (especially δ^18^O_b_) being more variable and including more outliers.

The model ensemble contains 3,000 different parametrizations for each region and water depth, picked semi-randomly from the three time slices and different combinations of modifications to incorporate variations of the model systematics, non-conservative proxy behaviour and the exclusion of individual proxies or source waters (Supplementary Table 6). We regard the final model ensemble as representative of a large range of potential past source water distributions that generally encompasses the limited knowledge about past non-conservative effects, sampling biases, source water properties and transient changes within each time period. From the whole ensemble we select the best 20% of model realizations in each time slice for the source water mixing estimates given in the main text and Figs. 3 and 4. See Extended Data Fig. 9 for a synthesis of the different mixing model results and Supplementary Figs. 4 and 5 for model quality assessment via Taylor diagrams.

### Validity of the mixing model

The principal validity of the multi-proxy mixing model was assessed via a direct comparison of its performance in estimating NADW abundance from oceanographic parameters (corrected for remineralization using the Redfield ratios given in ref. ^3^) with estimates from an optimized multi-parameter analysis (OMPA; Extended Data Fig. 5 and ref. ^3^). The direct comparison shows the very good agreement of both methods, but it also shows that extreme values (close to 0 or 100% NADW) agree less well, which presumably is rooted in the methodological differences between our Bayesian approach and the OMPA approach^86^. The mean absolute error between both methods for bulk NADW mixing fraction was 5%.

Another assessment of the validity of our approach in using a multi-proxy mixing model comes from the comparison between LH estimates of NADW abundance of 57% (95% range: 52–78%) compared to that from OMPA (75%; Extended Data Fig. 9). This comparison could indicate a bias towards low values in our proxy-based estimates. However, whether this bias is consistent between the Holocene and the two glacial time slices cannot easily be evaluated. Importantly, our LH estimates suffer from the fact that modern source water proxy signatures span only small ranges in proxy spaces, which decreases the quality of the source water mixing estimates (Extended Data Fig. 8). We thus focus our discussion on the comparison between the two glacial time slices, and note that the comparison with the Holocene and the absolute quantification of NADW abundance is less certain. Similarly, estimates of individual NSW abundances are less precise than for bulk NADW due to their similarity in proxy signatures, especially during the LH (Extended Data Fig. 8), whereas the quantification of bulk NADW (that is, the a posteriori combination of its tributary source waters) is considerably more precise.

### Fit quality of the mixing model

To evaluate the model ‘goodness of fit’, we rely on Taylor diagrams (Supplementary Figs. 4 and 5). To simplify the choice of best fitting models, we chose the 20% of model runs for each time slice that were closest to the observations in each Taylor diagram. The model errors are generally similar to those of the proxy observations themselves (Supplementary Figs. 6–8; note that in these figures some of the biases to high values in δ^13^C_b_, [CO_3_^2−^] and εNd are deliberately caused by the model modifications, as described in Supplementary Table 6).

### Source waters

The choice of relevant source waters and their characteristics, such as proxy signatures and concentrations, is decisive for the outcome of the proxy mixing model. Here we defined six source waters for the LGM and HS1 Atlantic, in addition to three source waters for the LH and modern (Figs. 1 and 2, Extended Data Fig. 8 and Supplementary Tables 4 and 5). Of the six glacial source waters, three have already been described in considerable detail in the literature (AABW, PDW and u-NADW), although not all relevant proxy signatures have been ascribed (see the main text and, for example, refs. ^16,18,21,22,34,36,40,68,72,87^). Source water u-NADW-2 has essentially been described in the same literature, although it was not generally considered to be an actual source water or was taken as glacial Antarctic Intermediate Water. Its characteristic deviations in proxy signatures from u-NADW-1 have been mostly explained by some combination of increased carbon remineralization, or meteoric water admixture, and warming, with the potential of additional SSW admixture^34,50,52,87^.

We estimated the remaining NSW carbon and oxygen isotope signatures as described in the main text, and the remaining proxy signatures from additional literature^14,35,40,88^ and glacial proxy data distributions (Extended Data Figs. 2 and 3). Radiocarbon ventilation ages for u-NADW and AMW are taken from the ‘mid-depth’ and ‘deep’ Atlantic data compilations from ref. ^88^. There is some potential for bias for the definition of some source water proxy signatures, in particular for the radiocarbon ages, [CO_3_^2−^] and εNd of the two AMW modes. We translate this into larger uncertainties of our proxy signature estimates, which are fully considered in the mixing calculations^14,76,89^ (Fig. 2). AMW εNd signatures are taken from a smoothed estimate of ref. ^14^. For the mixing calculations, we generally allow all source waters to contribute, that is, AABW, l-NADW and u-NADW for the modern and LH, and AABW, PDW, l-NADW-1, l-NADW-2, u-NADW-1 and u-NADW-2 for the LGM and HS1, albeit some source waters are removed by some model modifications (Supplementary Table 6 and Supplementary Figs. 14 and 15). Note that AABW and PDW were assigned slightly different proxy signatures for the LGM and HS1, as they are reflected by the defining proxy records in the abyssal South Atlantic and in the deep Pacific, respectively (Extended Data Fig. 2 and Supplementary Table 4). Radiocarbon ventilation ages for AABW and PDW are taken from the ‘deep’ Southern and Pacific Ocean data compilations in ref. ^88^.

Apart from the actual proxy signatures of the source waters, the concentrations with which the proxies are transported in the respective source waters affect the mixing results. This is particularly relevant for Nd, whose concentration in intermediate to deep waters today varies by roughly a factor of two and can presumably be affected by climatically induced changes in continental weathering^45,90^. To a much lesser degree it also affects DIC (the relevant concentration for δ^13^C_b_ and radiocarbon), which varies by roughly 10% in today’s open oceans. In addition, none of these concentrations can currently be directly reconstructed, and they are thus essentially unknown for the considered source waters, although several studies have estimated past DIC (for example, refs. ^22,91,92^). We therefore initially assume modern-like concentrations for all source waters. For DIC, we adopted the suggested concentrations for PDW, AABW and u-NADW from ref. ^22^. Furthermore, we suggest that the concentration of Nd in AMW is the least constrained, as the production of this source water and the weathering regime in its source region were presumably the most different from today^45^. Hence, we incorporated a series of modifications, varying the Nd concentration of AMW or alternatively equalizing the Nd concentration for all source waters (Supplementary Table 6).

The following nomenclature for the volumetric contributions of the different source waters is used in this study:

NADW = u-NADW + l-NADW = NADW-1 + NADW-2

u-NADW = u-NADW-1 + u-NADW-2

l-NADW = l-NADW-1 + l-NADW-2

NADW-1 = u-NADW-1 + l-NADW-1

NADW-2 = u-NADW-2 + l-NADW-2

AMW = AMW-1 + AMW-2

SSW = AABW + PDW

NADW + SSW = 1

See Supplementary Text 2 for a discussion of the connection between glacial AMW and l-NADW^8^.

## Online content

Any methods, additional references, Nature Portfolio reporting summaries, source data, extended data, supplementary information, acknowledgements, peer review information; details of author contributions and competing interests; and statements of data and code availability are available at 10.1038/s41561-025-01685-5.

## Supplementary information

Supplementary Information Supplementary Texts 1–3, Tables 1–7 and Figs. 1–18.

Supplementary Data 1 Supplementary Tables 8–12.

## Data Availability

All data used for Figs. 1 and 2 and for water mass mixing estimates are available via Zenodo at 10.5281/zenodo.14790596 (ref. ^93^).

## Code availability

The code of the Bayesian mixing model is provided by the published R package simmr^43^ and its application is described in the Methods and Supplementary Information.
