# Supplementary material for: Prevalent North Atlantic Deep Water during the Last Glacial Maximum and Heinrich Stadial 1
Source: Nat Geosci. 2025 May 6;18(5):410–6. doi: 10.1038/s41561-025-01685-5 (PMC12075000; doi:10.1038/s41561-025-01685-5)
Supplement: Supplementary file 1 — Supplementary Texts 1–3, Tables 1–7 and Figs. 1–18. [file 41561_2025_1685_MOESM1_ESM.pdf]

nature geoscience

Supplementary information https://doi.org/10.1038/s41561-025-01685-5

Prevalent North Atlantic Deep Water during the Last Glacial Maximum and Heinrich Stadial 1

In the format provided by the authors and unedited

Table of Contents

Supplementary Text 1: Proxies used in this study

Supplementary Text 2: Connection between AMW and l-NADW

Supplementary Text 3: Descriptions of specific glacial source waters

Supplementary Table 1: Regional salinity - oxygen isotope signature regressions from seawater.

Supplementary Table 2: Number of proxies per site in data compilation.

Supplementary Table 3: Number of sites per proxy in data compilation.

Supplementary Table 4: Estimated source water proxy signatures.

Supplementary Table 5: References for source water proxy signatures.

Supplementary Table 6: List of modifications for the applied mixing model.

Supplementary Table 7: Source water mixing estimates for the deep Atlantic during LH, HS1, and LGM.

Supplementary Table 8 (separate file): Carbon and oxygen stable isotope data used for Fig. 1.

Supplementary Table 9 (separate file): Data for source water proxy records used for Extended Data Fig. 2.

Supplementary Table 10 (separate file): Summary of sediment core sites including number of available data for each of the five proxies used.

Supplementary Table 11 (separate file): Full time period averaged proxy data set used for the mixing model calculations.

Supplementary Table 12 (separate file): Full list of runs and their modifications used for the mixing model ensemble.

Supplementary Figure 1: Sensitivity of $\delta^{18}O$ ice volume correction to age uncertainty.

Supplementary Figure 2: Linear regressions of $\delta^{18}O$ in seawater versus salinity for water depths > 200 m.

Supplementary Figure 3: Atlantic benthic $\delta^{18}O_b$ data vs. modern water depth, averaged for each site and ice volume corrected.

Supplementary Figure 4: Taylor diagrams showing summary statistics of the different mixing model runs of the depth-smoothed model.

Supplementary Figure 5: Taylor diagrams showing summary statistics of the different mixing model runs of the box-pooled model.

Supplementary Figure 6: Probability density distributions of depth-smoothed model proxy errors from observations in the best fit runs.

Supplementary Figure 7: Comparison of depth-smoothed model proxy signatures with actual observations for the Late Holocene.

Supplementary Figure 8: Comparison of depth-smoothed model proxy signatures with actual observations for Heinrich Stadial 1.

Supplementary Figure 9: Comparison of depth-smoothed model proxy signatures with actual observations for the Last Glacial Maximum.

Supplementary Figure 10: Performance of individual proxies to reconstruct source water contributions.

Supplementary Figure 11: NADW contributions different ensemble subsets.

Supplementary Figure 12: Source water fractions in the North Atlantic for different proxy combinations used, determined from the smoothed and interpolated data.

Supplementary Figure 13: Source water fractions in the South Atlantic for different proxy combinations used, determined from the smoothed and interpolated data.

Supplementary Figure 14: Source water fractions in the North Atlantic for different combinations of excluded source waters, determined from the smoothed and interpolated data.

Supplementary Figure 15: Source water fractions in the South Atlantic for different combinations of excluded source waters, determined from the smoothed and interpolated data.

Supplementary Figure 16: Comparisons of bulk NADW contributions in the box-pooled model during LGM and LH.

Supplementary Figure 17: Comparisons of bulk NADW contributions in the box-pooled model during HS1 and LGM.

Supplementary Figure 18: Mixing at abyssal north-west Atlantic Site KNR197-10-17.

Supplementary Texts

Supplementary Text 1: Proxies used in this study

In the following we give an overview of the five proxies used and discussed in the main text. We also briefly discuss the geochemical processes that are considered in the mixing model (see also Supplementary Table 6).

Stable carbon isotopes

Stable carbon isotopes have been most widely used for the reconstruction of deep water mass nutrient content and origin. The $\delta^{13}C$ of bottom water dissolved inorganic carbon (DIC) is archived in the carbonate shells of the epibenthic foraminifera genus *Cibicides* [1,2], preserved in marine sediments. The stable carbon isotope signature of seawater is affected by the degree and the temperature of CO_2 equilibration between air and surface waters, it is increased by photosynthesis, which discriminates against ^{13}C, and it is decreased by the remineralisation of organic matter, releasing ^{13}C depleted organic compounds to the DIC pool. Since the remineralisation of organic matter also releases the contained nutrients such as phosphate and others, $\delta^{13}C$ in the deep ocean anti-correlates with nutrient concentration. It can thus be used to distinguish the less ventilated and more nutrient rich AABW from the better ventilated and nutrient poor NADW in the modern ocean. The concentration of DIC in AABW and NADW is similar (~2250 vs. ~2140; [3,4] and thus the relationship of $\delta^{13}C$ versus % northern sourced water (NSW) is linear today.

The remineralisation of organic matter in the deep Atlantic mainly reduces reconstructed $\delta^{13}C$. We thus compensated for this effect virtually by increasing the $\delta^{13}C$ values of the observations by a variable degree (Supplementary Table 6). Furthermore, several records from the deep South Atlantic have been hypothesised to be influenced by either the "Mackensen Effect" or a "Habitat Effect" [5–7] leading to very low glacial $\delta^{13}C$ signatures. We thus incorporated the possibility to compensate for either of these effects individually in the mixing model.

Stable oxygen isotopes

The stable oxygen isotope signature of water is mainly affected by fractionation through evaporation (favouring ^{16}O) and precipitation (favouring ^{18}O). In marine surface waters it is thus determined by net evaporation (increasing $\delta^{18}O$ and salinity) and continental run-off such as river water (decreasing $\delta^{18}O$ and salinity), and hence correlates strongly with salinity. Additionally, sea ice formation in polar regions leads to salinification through brine rejection with insignificant isotopic fractionation [8]. Consequently, water that was salinified by brine rejection and which has the same salinity as a reference water that was salinified by evaporation will exhibit lower $\delta^{18}O$ signatures.

Oxygen atoms archived in foraminifera calcite are precipitated from seawater with additional temperature-dependent fractionation [9,10]. This leads to $\delta^{18}O$ correlating negatively with precipitation temperature. The combination of both fractionation effects leads to foraminifera calcite $\delta^{18}O$ correlating positively with water density, unless there is a significant contribution of brine-related salinification in the source water [8,11]. There are no other processes significantly affecting $\delta^{18}O_b$ in the deep ocean and it is thus a very conservative tracer.

The major challenges of using $\delta^{18}O$ is that its analysis is rather imprecise (analytical uncertainty in terms of single standard deviation is usually around 0.07 ‰) and that uncorrected gas mixing in the mass spectrometer source or other non ideal instrument performance may lead to inter-laboratory offsets of up to 0.30 ‰ [12]. Considering that the range of $\delta^{18}O$ in sediment core top benthic foraminifera ($\delta^{18}O_b$) in the Atlantic spans roughly 0.80 ‰ these uncertainties limit the use of $\delta^{18}O$ as a source water tracer today. However, since the range of observed $\delta^{18}O_b$ in the glacial is much larger (> 2 ‰, see Supplementary 1), it is actually a valuable tracer for the LGM and HS1.

In the mixing model we therefore did not use $\delta^{18}O_b$ as a source water proxy for the modern or Late Holocene cases, and we corrected $\delta^{18}O_b$ signatures for the two glacial time slices. Since $\delta^{18}O_b$ is

precipitated from water molecules, its concentration in source waters is infinite and more importantly exactly equal for all source waters.

Carbonate ion concentration

Boron concentrations in benthic foraminifera tests correlate linearly with local carbonate ion concentration [CO_3^{2-}] in seawater (with species-specific correlation equations; [13]. Seawater [CO_3^{2-}] in turn depends on seawater pH, alkalinity, and DIC concentrations, and very little on seawater temperature [14]. In effect, air-sea CO_2 exchange, alkalinity changes in surface waters, and remineralisation of organic matter are the major determinants of source water [CO_3^{2-}], and only remineralisation affects subsurface waters (Supplementary 2), and in a predictable ratio compared to the changes it induces in $\delta^{13}C$ [14].

In the model we corrected for the imprint of organic matter remineralisation on [CO_3^{2-}] in the same way as for stable carbon isotopes, applying a conversion factor of 43 µmol/kg per 1‰ change applied to $\delta^{13}C$ following [14].

Radiocarbon ventilation age

Radioactive ^{14}C is produced by cosmic rays in the upper atmosphere, mixed into the atmospheric carbon pool, and enters seawater through gas exchange, while it decays radioactively with a half live of about 5.7 ka. Notably, the isotopic equilibration time of radiocarbon between seawater and atmosphere is more than 10 years and thus very long compared to gas exchange times of CO_2 in the order of months [15]. In deep waters, the radiocarbon isotopic signature is mainly changed by radioactive decay and hence ageing of a water parcel. Here we exclusively consider the differences between radiocarbon ages from measurements of benthic samples to the contemporary atmosphere, $^{14}C_{b\text{-}atm}$, i.e. the radiocarbon ventilation age [16].

Within the modern Atlantic, actual deep water ageing is less than 500 years [17,18], but could well have been larger during the LGM and in particular during HS1. However, a reasonable implementation of

a correction for deep water ageing would require knowledge of advection speeds and pathways, which is beyond the simple mixing model employed here. Considering the large range (400 – 3000 years) and uncertainties (800 – 1200 years; 2 standard errors) in our assigned glacial source water radiocarbon ages we suppose that the effect of ageing within the deep Atlantic is not a dominant control on glacial Atlantic deep water radiocarbon signatures, however, and did not implement it as a specific parametrisation.

Radiogenic neodymium isotopes

Neodymium is a rare earth element and a trace metal in the environment. It is primarily added to seawater through continental run-off, dissolution of dust in the sea surface, and dissolution of particles in marine sediments. Different rocks carry different radiogenic Nd isotopic signatures (εNd) which are imprinted into source waters by these processes. Dissolved Nd is rather particle reactive and hence adsorbs to sinking particles in the water column while being advected with seawater [19].

In the modern Atlantic with its vigorous large scale circulation, advection is fast enough to transport Nd across basins before the effect of sinks and sources becomes dominant, so that it behaves largely conservative in deep waters. However, the sinks and sources may have changed through time, in particular on glacial – interglacial time scales, which can affect the isotopic signatures of source waters as well as the conservativeness of these signatures during water mass advection in the Atlantic [20,21].

Furthermore, the concentration of Nd in different source waters varies substantially, in the modern analogues to the ones used here roughly by a factor of two from 18 pmol/kg in u-NADW to 35 pmol/kg in PDW. There is currently no method of reconstructing past seawater Nd concentrations directly and the rather complex interplay of sources and sinks make it rather complex to estimate past source water Nd concentrations [21].

Lastly, the integrity of sedimentary archives of past deep water εNd is still debated and often questionable [19]. For example, sedimentary authigenic εNd from the Northwest Atlantic and Labrador Sea have been shown to be less radiogenic than local bottom water, apparently due to exchange of Nd with pore waters, which incorporate Nd from partial dissolution of lithogenic sediments [22,23]. But again, it is not certain to which degree these effects occurred in the glacial environment as well.

In the mixing model we incorporate three different parametrisations for the different Nd isotope systematics, in order to simulate the effects of different source water Nd concentrations, fluxes of (unradiogenic) Nd from sediments into southern sourced water (SSW) within the Atlantic, and biased Northwest Atlantic and Labrador Sea εNd records (see Supplementary Table 6).

Supplementary Text 2: **Connection between AMW and l-NADW**

AMW presents the most important source for lower NADW today. Its overflow across the Greenland Scotland Ridge leads to strong entrainment so that, for example, Denmark Strait Overflow Water exhibits a fractional entrainment factor of 0.55, meaning it is composed of 45 % AMW and 55 % upper NADW and shallow waters entrained into the overflow [24].

For consistency, we used the source water type definitions of ref [25] for modern and Late Holocene source waters, which comprises l-NADW, but not pure AMW before entrainment. On the other hand, our method of identifying source water signatures in the form of corners in mixing polygons of proxy-proxy plots such as Fig. 1 should yield the signatures of AMW, if it is covered by the data. Assuming u-NADW is the source water that is mostly entrained by AMW, then l-NADW should lie within the polygon of NADW source waters. For the multi-proxy mixing calculations we thus used l-NADW for the Late Holocene case and glacial AMW for the LGM and HS1 cases.

The process of source water signature definitions for the glacial time periods is associated with significant uncertainties, mainly because it is not clear whether the proxy signatures we assign to northern sourced waters are pure or are already a mixture of source waters. Similarly, it is not

entirely clear whether the proxy signatures assigned to AMW should rather be assigned to the already mixed l-NADW. This would make the overall ratio of AMW and u-NADW in the glacial Atlantic even more similar to today. Nonetheless, the spatial closeness of carbon and oxygen isotope signatures of AMW-1 and those found in the deep Irminger and Labrador Sea (Fig. 1) suggests that glacial l-NADW contained a larger fraction of AMW than today, i.e. that entrainment was reduced.

It is important to note that the related uncertainties do not affect the calculations for the overall amount of NADW in the glacial Atlantic, since both AMW and l-NADW are components of NADW. Further multi-proxy studies at individual sites as close as possible to the different source water regions would be necessary in order to better define the different source water proxy signatures and to decipher the connection between u-NADW, l-NADW, and AMW more precisely.

Another interesting feature is that the suggested source water AMW-2 is not actually observed in the glacial proxy data from the Arctic Mediterranean (Fig. 1). This could indicate that AMW-2 did not exist in the way we suggest. For example, the stable isotope signatures trending towards the values of AMW-2 could be formed by mixing u-NADW-2 into a mixture of AMW-1, u-NADW-1, and SSW. Alternatively, AMW-2 could have been formed from AMW-1 within the western subpolar North Atlantic, for example by sea ice brine rejection along the Greenland and Labrador shelves (see also refs [26, 27]), or subsurface mixing of source waters. Detailed local multi-proxy investigations would be necessary to get more certainty. The effect of removing AMW-2 from the mixing calculations is negligible, changing the average NADW contributions in the deep Atlantic during both the LGM and HS1 by only 1 % (see Supplementary Data Figs. 11, 14 & 15).

Supplementary Text 3: Descriptions of specific glacial source waters

Pacific Deep Water

Deep Atlantic SSW today is composed of AABW, whereas in the glacial PDW apparently also contributed [7]. The proxy signatures between these two SSW are generally more similar than they are

to the four tributaries of NADW, therefore making our *a posteriori* calculations of SSW and NADW more robust and precise than those for individual source waters (Fig. 3 & Extended Data Fig. 9). While the source water attribution to the two SSW types is hence less precise, the mixing model ensemble results do indicate several trends that differentiate these two source waters (Fig. 3). For example, AABW occupied deeper layers and hence was clearly more dense than PDW, which is similar to today and in agreement with [7]. In the South Atlantic AABW was most prevalent below 4 km depth and PDW was layered on top as shallow as 3 km water depth. On their advance into the North Atlantic both SSWs mixed with NADW, reducing their fractional abundance in the North Atlantic, especially at ~ 3 km water depth. The advance of PDW into the (north) Atlantic presumably increased the global volume of this carbon rich water mass, increasing carbon sequestration from the atmosphere into the deep ocean [7]. Data from the South Atlantic could indicate that PDW was already partially replaced by AABW during HS1, which would support the rise of atmospheric CO_2 concentrations at that time (Fig. 3).

Our identification of PDW as source water essentially follows ref. 28. It is particularly well identified by its low carbonate ion concentration of ~ 50 ± 10 µmol/kg and its high εNd signatures of -5 ± 0.5, and is particularly prevalent in the deep South East Atlantic at around 4 ± 0.5 km water depth during the LGM. However, our analysis does not directly indicate how this source water is formed. If there was no substantial deep water formation in the LGM Pacific, we can assume that most Pacific deep waters originated in the Circumpolar Current similar to today, i.e. they were a mix of Antarctic Bottom Water – particularly the type formed in the Ross Sea – and NADW. Slow circulation through the deep Pacific would then have aged the water mass similar as it does today, explaining its high radiocarbon age and low carbonate ion concentration.

North Atlantic Bottom Water

It has been hypothesised that the abyssal North Atlantic saw a very dense North Atlantic Bottom Water (NABW) during the LGM [29,30]. These studies were based on sediment core KNR197-10-

17GGC from Corner Rise in the northern Northwest Atlantic just South of the Labrador Sea at 5010 m water depth. While we did not include a dedicated NABW in our source water ensemble due to the lack of assured observations of this source water, the mixing model analysis does indicate that an AMW-like water was prevalent and protruded below or into AABW during the LGM, although much less so during HS1 (Fig. 3 & Supplementary Fig. 18). Potentially, this NABW was a particularly dense mode of AMW that allowed it to sink below AABW.

Supplementary Tables

Supplementary Table 1: *Regional salinity - oxygen isotope signature regressions from seawater. S = salinity in PSU. Equations yield seawater $\delta^{18}O$ vs. VSMOW in ‰.*

basin	water depth	equation
North Atlantic	< 2000 m	0.766 * S - 26.549
North Atlantic	>= 2000 m	1.409 * S - 48.950
South Atlantic	< 2000 m	0.488 * S - 16.859
South Atlantic	>= 2000 m	1.572 * S - 54.649
Pacific	all	0.4385 * S - 15.1851
Arctic Mediterranean	< 1500 m	0.3654 * S - 12.485

Supplementary Table 2: *Number of proxies per site used for the estimation of NADW contributions.*

# of proxies:	1	2	3	4	total sites
LH	13	48	12	1	74
HS1	9	61	11	2	83
LGM	16	60	16	4	96

Supplementary Table 3: *Number of sites for each proxy used for the estimation of NADW contributions. Number in brackets indicates sites with calculated Late Holocene offset to local seawater.*

proxies:	$\delta^{13}C$	$\delta^{18}O$	$[CO_3^{2-}]$	εNd	^{14}C
LH	62	54 (39)	7	19	7
HS1	73	70 (40)	1	15	13
LGM	79	71 (43)	10	21	19

***Supplementary Table** 4: Estimated source water proxy signatures.*

source water	used for time slice	$\delta^{13}C_b$ (‰)	$\delta^{18}O_b$ (‰) (ivoc)	$[CO_3^{2-}]$ (µmol/kg)	εNd	$^{14}C_{b-a}$ age (years)	[Nd] (pmol/kg)	DIC (µmol/kg)
**modern:**								
**AABW**		0.40 ± 0.05	3.20 ± 0.05	83 ± 5	-8.5 ± 0.5	1500 ± 150	26.5 (1.00)	2250 (1.00)
**u-NADW**		1.30 ± 0.05	2.70 ± 0.10	120 ± 5	-14.2 ± 0.2	600 ± 150	18.3 (0.69)	2140 (0.95)
**l-NADW**		1.30 ± 0.05	3.25 ± 0.08	120 ± 5	-12.4 ± 0.4	1000 ± 150	22.8 (0.86)	2140 (0.95)
**glacial:**								
**AABW**	LGM	-0.90 ± 0.10	3.7 ± 0.10	87 ± 4	-6.8 ± 0.4	22500 ± 200	26.5 (0.76)	2400 (0.92)
	HS1	-0.70 ± 0.10	3.8 ± 0.10	87 ± 4	-7.3 ± 0.6	1750 ± 300	26.5 (0.76)	2400 (0.92)
**PDW**	LGM	-0.40 ± 0.10	3.30 ± 0.15	50 ± 10	-5.0 ± 0.5	3000 ± 400	35.0 (1.00)	2600 (1.00)
	HS1	-0.40 ± 0.10	3.30 ± 0.15	68 ± 10	-5.0 ± 0.5	2500 ± 400	35.0 (1.00)	2600 (1.00)
**u-NADW-1**	LGM & HS1	1.55 ± 0.05	3.72 ± 0.09	142 ± 10	-9.5 ± 0.7	1250 ± 200	18.3 (0.52)	2200 (0.85)
**u-NADW-2**	LGM & HS1	0.40 ± 0.10	2.20 ± 0.21	115± 10	-10.0 ± 1.0	1400 ± 250	18.3 (0.52)	2200 (0.85)
**AMW-1**	LGM & HS1	0.69 ± 0.10	4.00 ± 0.07	150 ± 10	-15 ± 1.5	2150 ± 500	22.8 (0.65)	2200 (0.85)
**AMW-2**	LGM & HS1	0.03 ± 0.12	2.90 ± 0.12	150 ± 20	-16 ± 1.5	2550 ± 500	22.8 (0.65)	2200 (0.85)

Estimated proxy signatures and concentrations of Nd and DIC of all source waters used. Numbers in brackets for concentrations are relative concentrations used in the mixing model.

***Supplementary Table** 5: References for source water proxy signatures*

source water	$\delta^{13}C_b$	$\delta^{18}O_b$ (‰)	$[CO_3^{2-}]$	εNd	$^{14}C_{b-a}$ age
**modern:**					
**AABW**	Yu et al. (2020)	Hoffman & Lund (2012)	Yu et al. (2020)	Yu et al. (2020)	Skinner et al. (2017)
**u-NADW**	Yu et al. (2020)	Hoffman & Lund (2012)	Yu et al. (2020)	Van de Flierdt et al. (2016)	Skinner et al. (2017) and seawater data
**l-NADW**	Yu et al. (2020)	Hoffman & Lund (2012)	Yu et al. (2020)	Lambelet et al. (2016)	Skinner et al. (2017) and seawater data
**glacial:**					
**AABW**	Sites TN057-21 & RC11-83 and Yu et al. (2020)	Sites TN057-21 & RC11-83	Site TN057-21 and Yu et al. (2020)	Sites TN057-21, ODP 1089 & RC11-83 and Yu et al. (2020), modern concentrations	Rafter et al. (2022) Southern Ocean
**PDW**	Sites RC23-15 & RC23-22, SO213/2 59-1 & SO213/2 60-2 and Yu et al. (2020)	Sites RC23-15 & RC23-22, SO213/2 59-1 & SO213/2 60-2	Sites RC23-15 & RC23-22 and Yu et al. (2020)	Sites SO213/2 59-1 & SO213/2 60-2 and Yu et al. (2020), concentrations related to LGM eNd offset from modern, see Howe et al. (2016)	Rafter et al. (2022) deep Pacific
**u-NADW-1**	Sites EW9302-24/25/26 GGC; see Fig. 1 and main text	Sites EW9302-24/25/26 GGC; see Fig. 1 and main text	Site BOFS 17 and Yu et al. (2008)	Sites BOFS 17 & KNR198-35GGC, modern concentrations	Rafter et al. (2022) Atlantic mid-depth
**u-NADW-2**	Sites EW9302-24/25/26 GGC; see Fig. 1 and main text	Sites EW9302-24/25/26 GGC; see Fig. 1 and main text	Site BOFS 17 and Yu et al. (2008)	Sites BOFS 17 & KNR198-35GGC, modern concentrations	Rafter et al. (2022) Atlantic mid-depth
**AMW-1**	Sites ODP 980 & SU90-24; see Fig. 1 and main text	Sites ODP 980 & SU90-24; see Fig. 1 and main text	Site ODP 980 and Yu et al. (2008)	Site IODP U1302 and Blaser et al. (2020), modern concentrations	see Extended Data Fig. 3
**AMW-2**	Sites ODP 980 & SU90-24; see Fig. 1 and main text	Sites ODP 980 & SU90-24; see Fig. 1 and main text	Site ODP 980 and Yu et al. (2008)	Site IODP U1302 and Blaser et al. (2020), modern concentrations	see Extended Data Fig. 3

Supplementary *Table 6:* *List of modifications for the applied mixing model. Modifications from each row were randomly combined with each other, and 3000 combinations were sampled for the model ensemble.*

model modification	implementation	values in ensemble	number of occurrences in ensemble
average observations	Average observations for each proxy in each box to one median value in order to remove bias from the number of data per proxy and box (not relevant for depth-smoothed approach).		486
modern distributions as prior	Use the modern distributions of AABW, l-NADW, and u-NADW as prior distributions for AABW and PDW, l-NADW, and u-NADW, respectively.		437
remove proxy from model	Remove certain proxies from mixing calculations (observations and source waters), ignoring them completely.	each proxy individually, $\delta^{18}O$ and εNd, or all carbon related proxies	1346
remove source water uncertainty	Set uncertainty of all source water signatures (see Supplementary Table 3) to zero.		421
remove AMW from model	Remove both AMW-1 and AMW-2 completely from potential source waters.		457
remove AMW-2 from model	Remove only AMW-2 completely from potential source waters.		323
remove mode-2 source waters from model	Remove both u-NADW-2 and AMW-2 completely from potential source waters.		331
correct for organic matter remineralisation	Remineralising organic matter exported from the ocean surface adds low $\delta^{13}C$ carbon to the deep DIC pool. We account for this process virtually by adding x ‰ to $\delta^{13}C_b$ observations at depths > 3 km, and 2/3 * x ‰ is added to observations between 2 and 3 km, roughly following the profile of remineralised $\delta^{13}C$ from Oppo et al. (2018) for the LGM. Furthermore, corresponding changes of[CO_3^{2-}] of +43 µmol/kg per ‰ change in $\delta^{13}C_b$ are applied to the same locations, following Yu et al. (2008).	x = {0, 0.1, 0.2, 0.3, 0.4}	1250
correct very low glacial South Atlantic $\delta^{13}C$ observations	In the glacial abyssal South Atlantic, very low $\delta^{13}C_b$ around -1‰ have been argued to not represent ambient seawater. This is corrected for following Yu et al. (2020) by adding x ‰ to the $\delta^{13}C_b$ of glacial AABW (correcting for a habitat effect), or alternatively adding x ‰ to the $\delta^{13}C_b$ and additionally +43 µmol/kg per ‰ of $\delta^{13}C_b$ to its [CO_3^{2-}] (correcting for the Mackensen effect). In both cases, observations of $\delta^{13}C_b$ < -0.75 ‰ are excluded from the mixing calculations.	x = {0, 0.25, 0.5}	1068
test with different Nd concentrations	The concentration of Nd in past source waters is unknown, and may have been significantly different. This counts in particular for AMW, which was probably the most different from modern analogues and additionally in contact with sediments in the Labrador Sea (Pöppelmeier et al., 2022). The concentration of Nd in AMW is increased by a factor of x, or alternatively Nd concentrations of all source waters are set equal.	x = {1, 1.5, 2, 2.5}	1315
correct for benthic flux of Nd	Benthic flux of Nd affects water masses in contact with sediment. This is particularly important for abyssal SSW, which is rendered less radiogenic on its northward advance. Hence, εNd of glacial PDW and AABW is decreased by x epsilon units in equatorial, north, and subpolar Atlantic model regions. South Atlantic regions are assumed to be not altered as they represent the actual source waters.	x = {0, 1, 2, 3, 4, 5}	1105
bias in Northwest εNd records	In Holocene sediments, εNd from the deep North American Basin and Labrador Sea is systematically less radiogenic than local bottom water, and it is uncertain to which degree such an effect also occurred during glacials (Pöppelmeier et al., 2019):<br>The εNd of observations in the NW Atlantic and depths >= 3 km and of northern sourced deep waters is increased by x. Alternatively, the εNd of AMW is increased by 3 units, assuming that reconstructions from the Labrador Sea were biased (Blaser et al., 2020).	x = {0, 1, 3, 5}	1372

Supplementary Table 7: Source water mixing estimates for the deep Atlantic during LH, HS1, and LGM as shown in Extended Data Fig. 9. *Numbers are medians, in per cent with 95 % prediction intervals.*

**time**	**NADW**	**u-NADW**	**l-NADW & AMW**	**NADW-2**	**AABW**	**PDW**	**number of runs**
**LH**	57.2 ± 12.7	38.4 ± 12.0	19.6 ± 21.0	-	42.8 ± 12.7	-	405
**HS1**	53.3 ± 10.1	27.6 ± 6.7	24.9 ± 12.8	23.7 ± 8.2	19.0 ± 5.6	27.3 ± 11.4	1293
**LGM**	51.7 ± 9.8	30.4 ± 11.6	19.9 ± 13.1	8.8 ± 3.5	17.7 ± 8.5	29.4 ± 8.2	1302

Supplementary Table 8 (separate spreadsheet): *Carbon and oxygen stable isotope data used for Fig. 1.*

Supplementary Table 9 (separate spreadsheet): *Data for source water proxy records used for Extended Data Fig. 2.*

Supplementary Table 10 (separate spreadsheet): *Summary of sediment core sites including number of available data for each of the five proxies used.*

Supplementary Table 11 (separate spreadsheet): *Full time period averaged proxy data set used for the mixing model calculations.*

Supplementary Table 12 (separate spreadsheet): *Full list of runs and their modifications used for the mixing model ensemble.*

Supplementary Figures

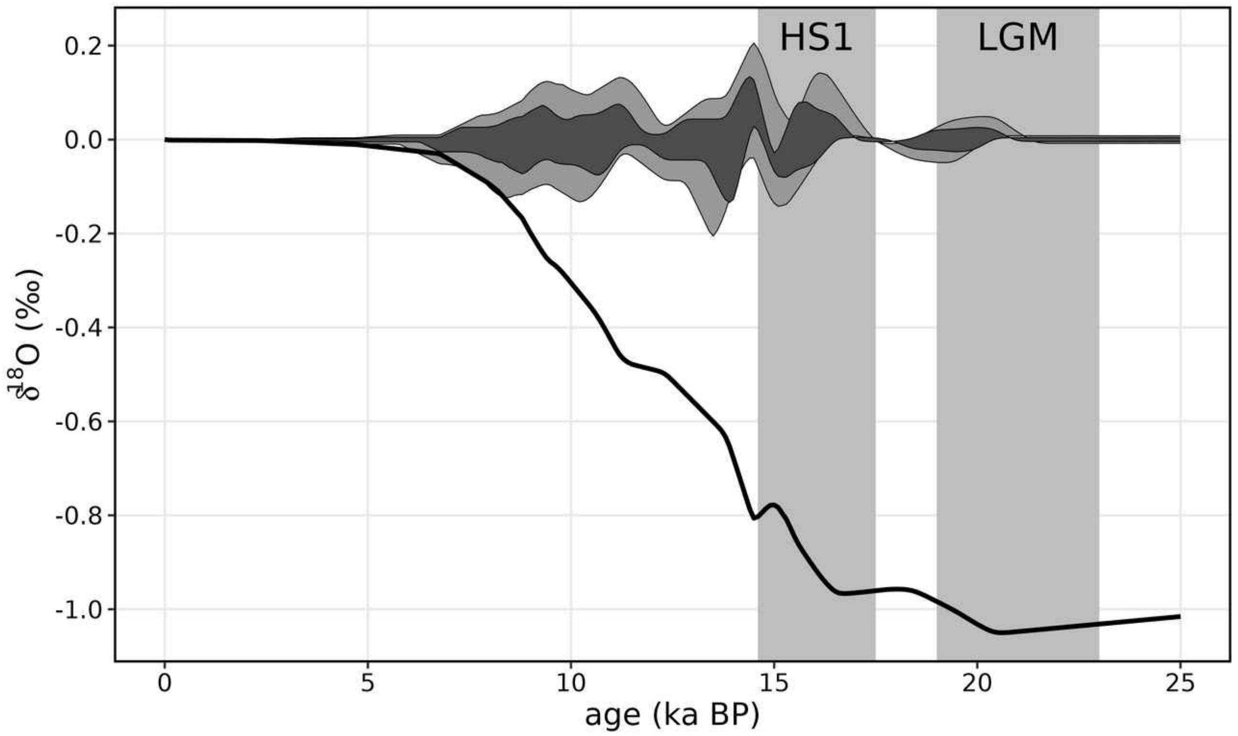

Supplementary Figure 1: *Sensitivity of $\delta^{18}O$ ice volume correction to age uncertainty. Black curve shows the correction curve applied, based on sea level reconstructions from ref* [31]*. Bright and dark grey bands are correction biases for age errors of 1.0 and 0.5 ka, respectively. Typical uncertainties of $\delta^{18}O$ analyses are around 0.15 ‰ (double standard deviation).*

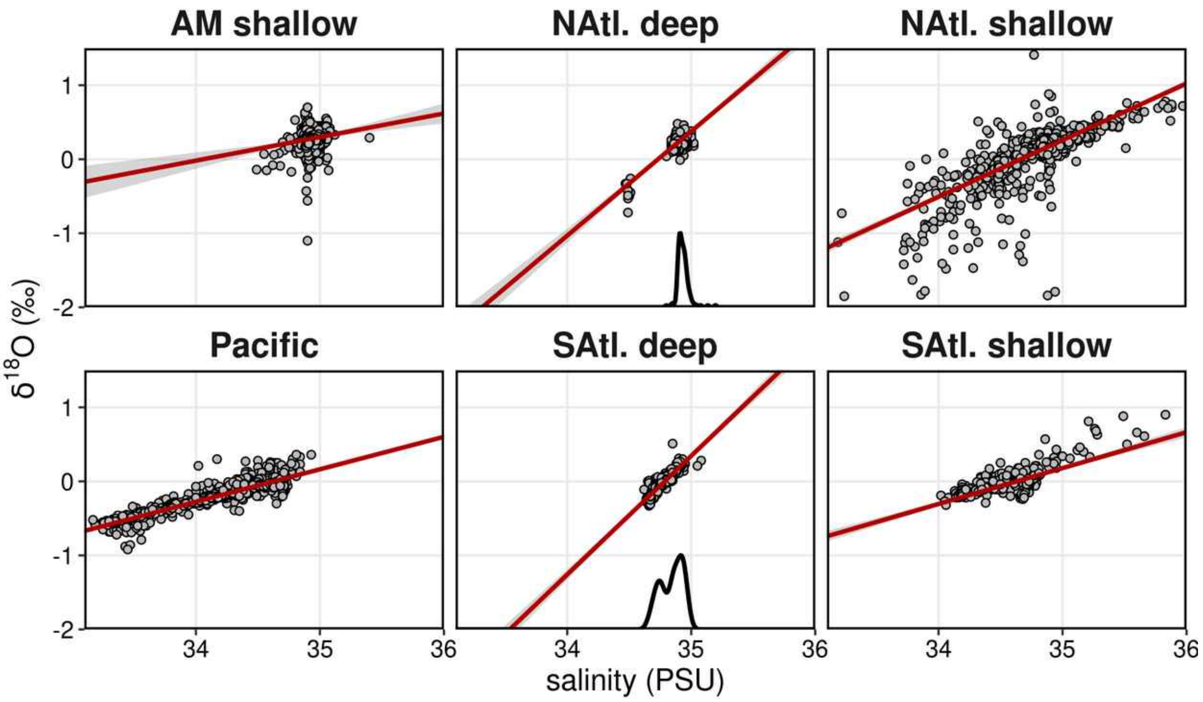

Supplementary Figure 2: *Linear regressions of $\delta^{18}O$ in seawater versus salinity for water depths > 200 m. The panels differentiate different spacial domains. In the central column, black curves indicate probability density distributions of modern salinity at sites contained in the LGM and HS1 data sets for the mixing model (on arbitrary y-axis). See Supplementary Table 1 for regression equations.*

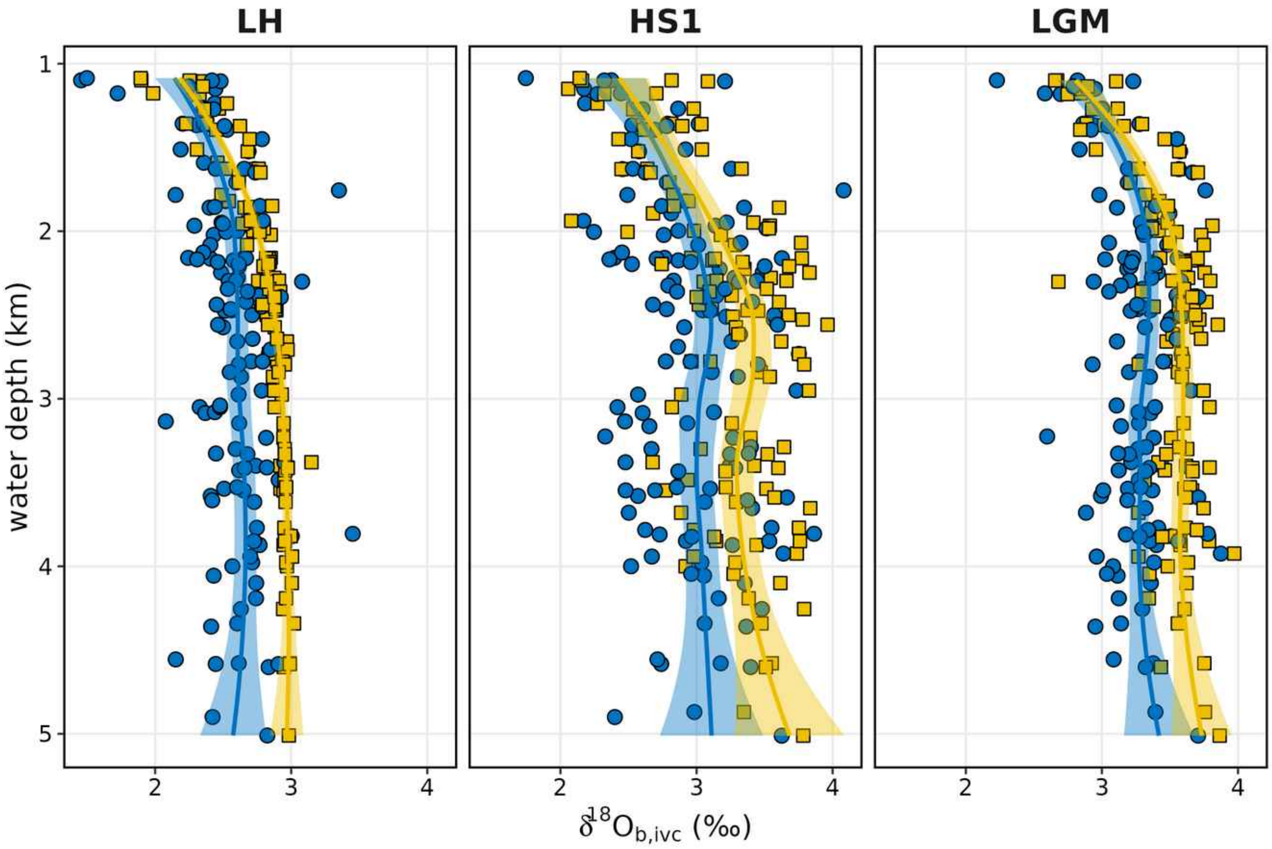

Supplementary Figure 3: *Atlantic benthic $\delta^{18}O_b$ data vs. modern water depth, averaged for each site and ice volume corrected. Colours denote data without further correction (blue) and with additional site-specific correction for the offset between Late Holocene data and local seawater (yellow). Lines and shading are LOESS smooths and their confidence intervals.*

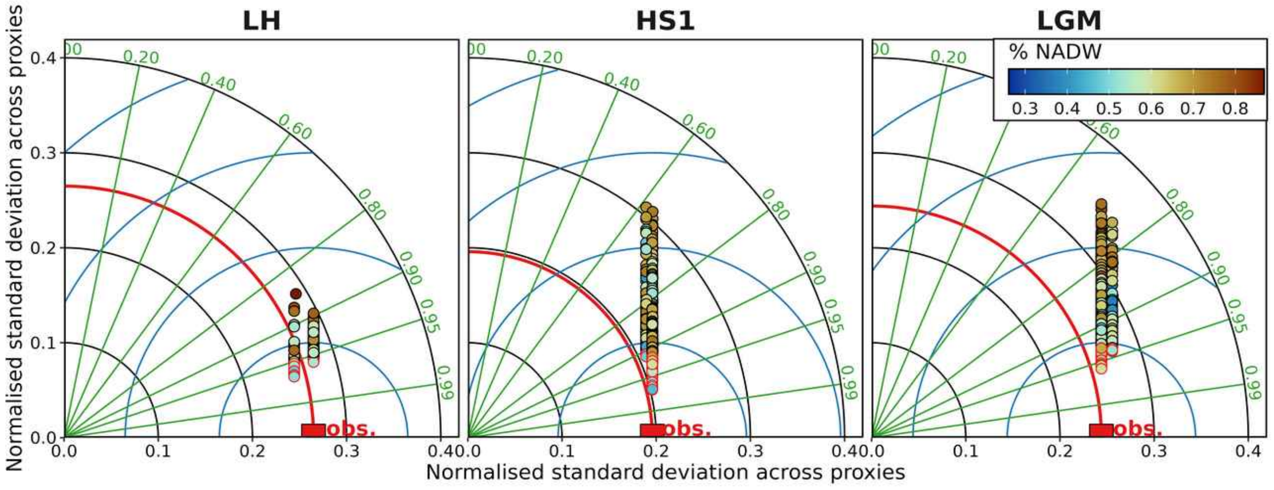

Supplementary Figure 4: *Taylor diagrams showing summary statistics of the different mixing model runs of the depth-smoothed model. Taylor diagrams show how closely the gross pattern of the modelled data match the pattern of the observations (red square). The distance from origin (black circles) indicates the relative standard deviation of the model results, green radial lines indicate correlation coefficient between observations and model results, blue circles centred on observations indicate root mean square error among observations and model results. The closer a simulation lies to the observations the better its pattern matches with those of the observations. The point colour indicates the fraction of NADW in the deep Atlantic in this specific model run. Red-bordered symbols are the 20 % of ensemble results closest to observations (see Methods).*

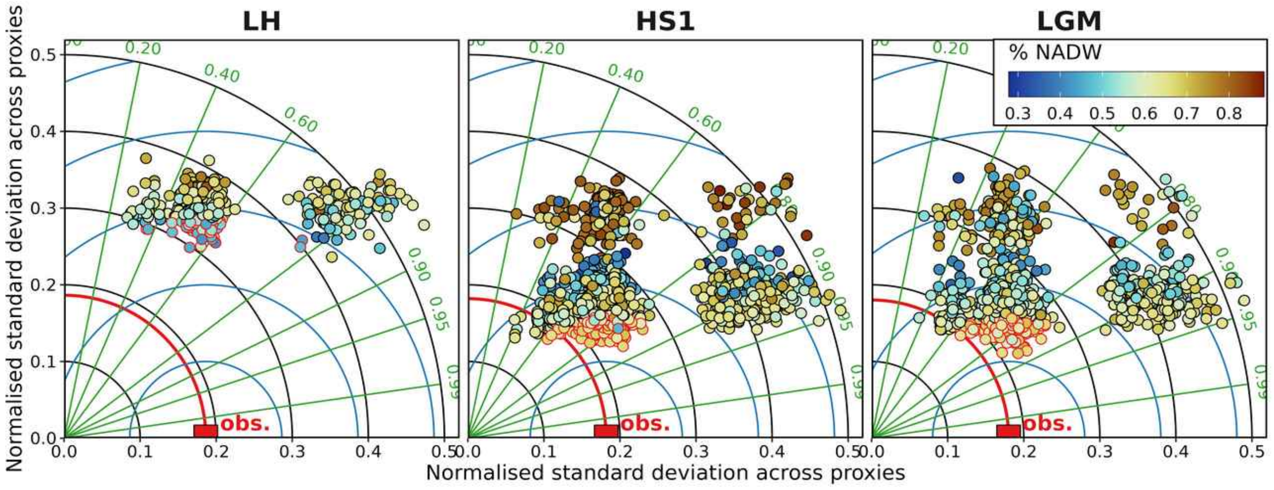

Supplementary Figure 5: *Same as Supplementary Fig. 4, but for mixing model runs based on box-pooled proxy data.*

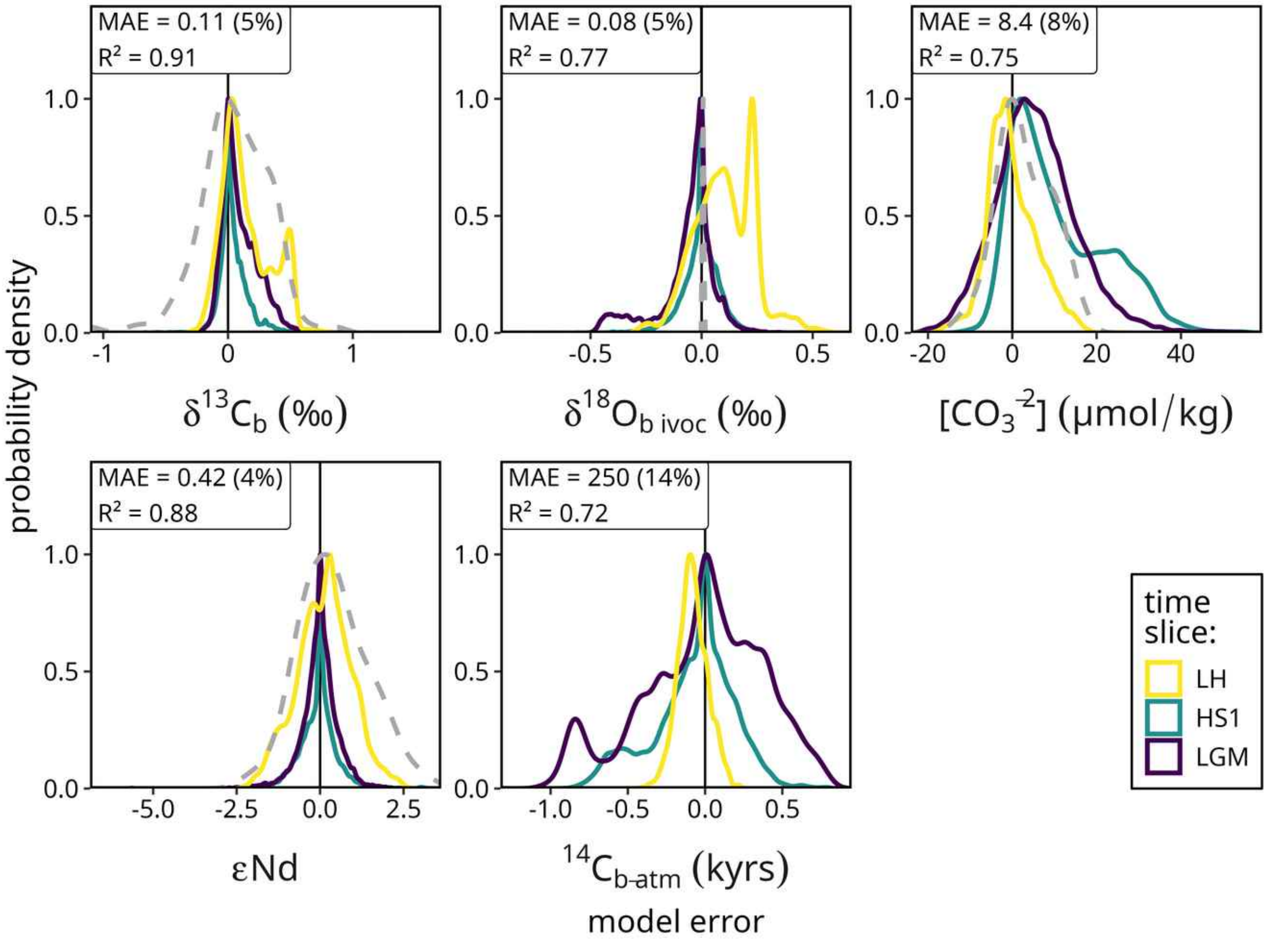

Supplementary Figure 6: *Probability density distributions of depth-smoothed model proxy errors from observations in the best fit runs. Labels show mean absolute errors in proxy units and relative to the range of proxy values in the glacial source waters (in brackets), and R^2 of linear regressions between modelled and observed data. Probability densities are scaled to 1 for better visibility. Dashed lines indicate errors between proxy signatures in Late Holocene sediment observations and estimated local seawater (equal to zero per definition for $\delta^{18}O_{b,ivoc}$). Note that some biases to high values in $\delta^{13}C_b$, $[CO_3^{2-}]$, and εNd are deliberately caused by the model modifications (see Supplementary Table 6).*

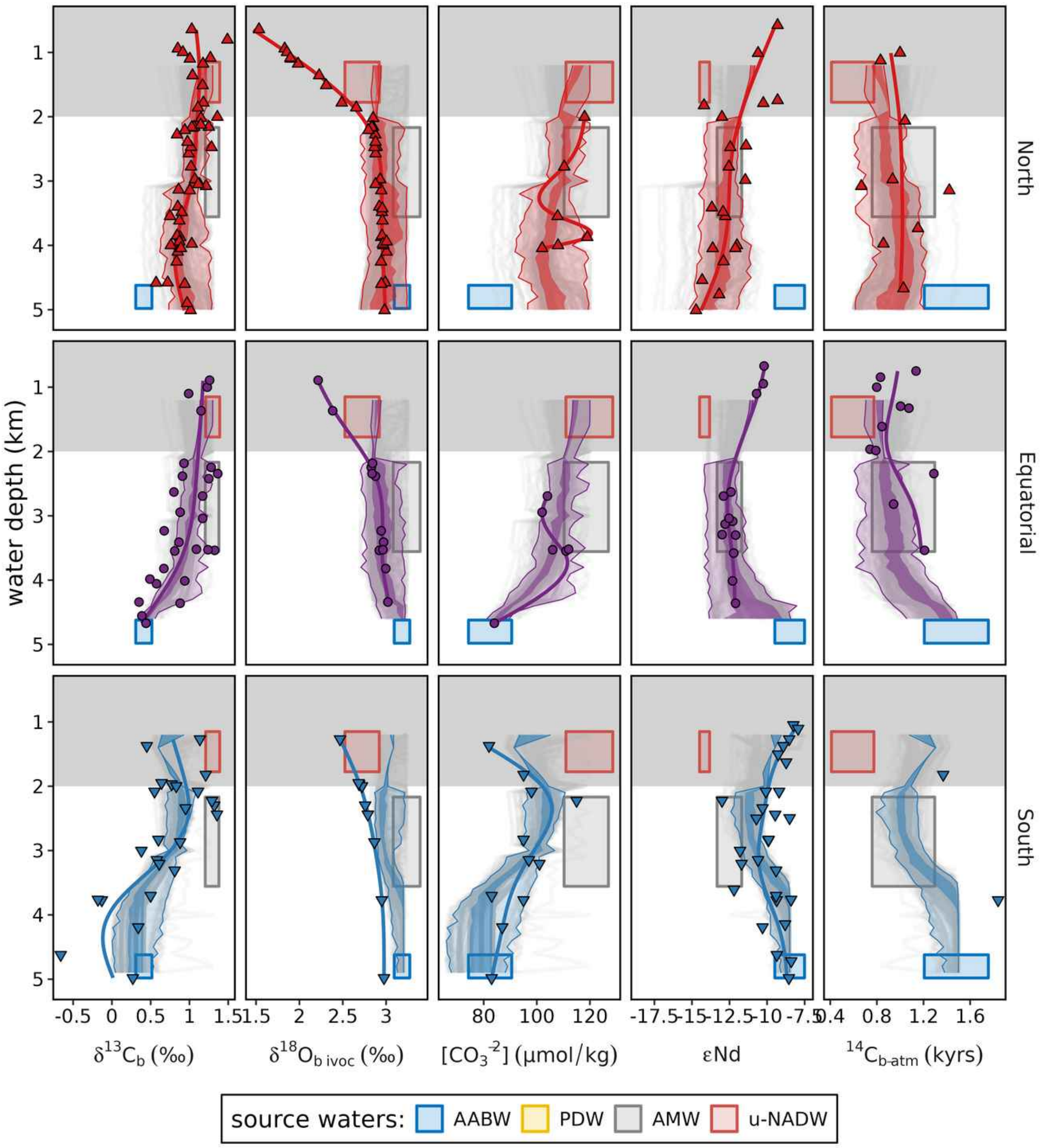

Supplementary Figure 7: *Comparison of depth-smoothed model proxy signatures with actual observations for the Late Holocene. Proxy reconstructions are shown as symbols and depth-smoothed proxy trends as thick lines (as in Extended Data Fig. 6). Thin grey lines in the background show the results of all individual mixing model runs, whereas the dark and bright shaded areas indicate central 50 % and 95 % of the best 20 % mixing model runs. Grey area at < 2 km depth indicates the depth range where the mixing model is less accurate due to the contribution of additional source waters. Coloured rectangles mark the proxy values of the different source waters across the depths at which they were defined. Note that PDW is only considered for the glacial runs (Supplementary Figures 8 & 9) and was defined from records in the Pacific (see Extended Data Fig. 2).*

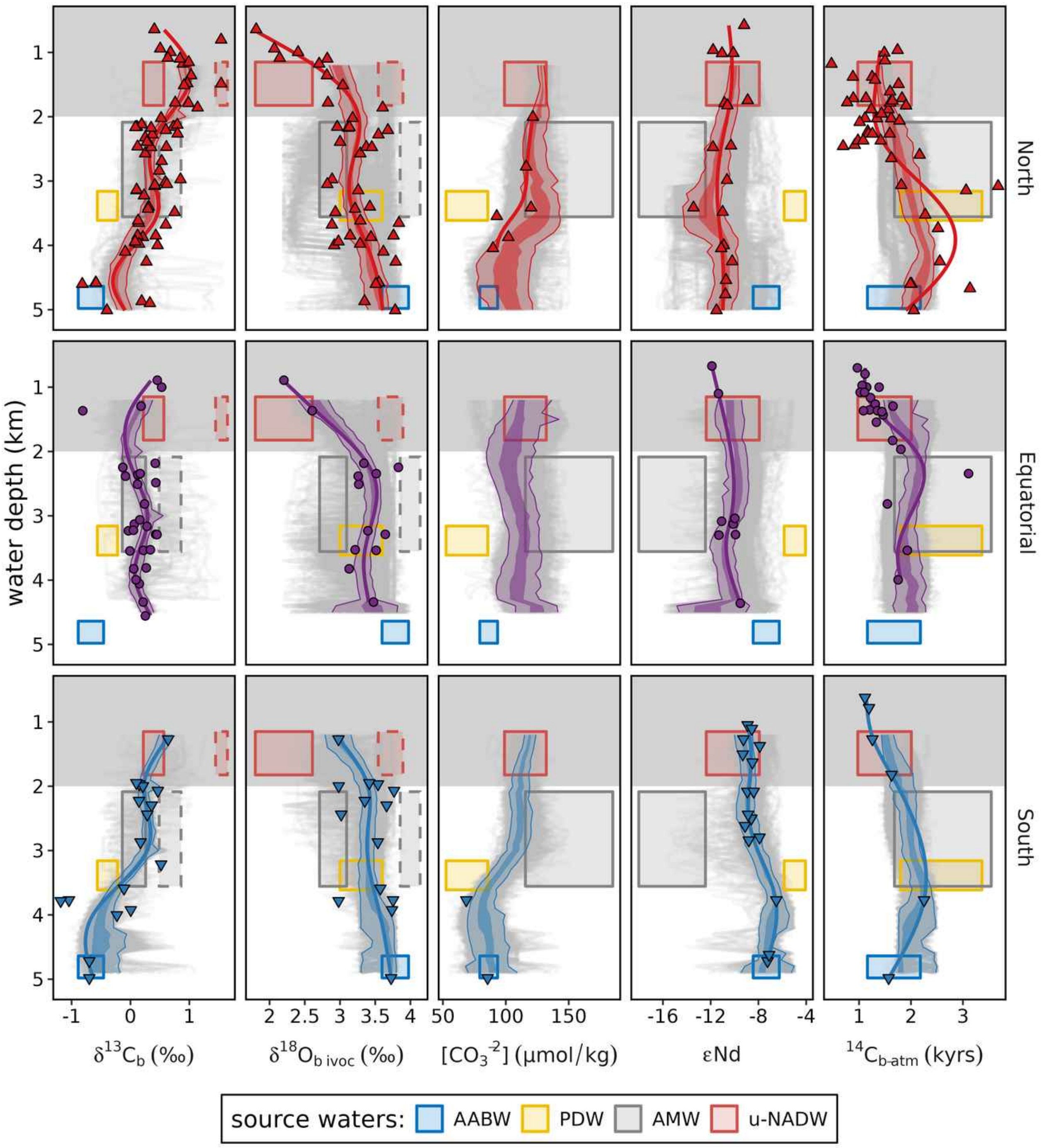

Supplementary Figure 8: *Same as Supplementary Fig. 7, but for Heinrich Stadial 1. Dashed rectangles indicate carbon and oxygen isotope signatures of mode 1 NADW source waters*

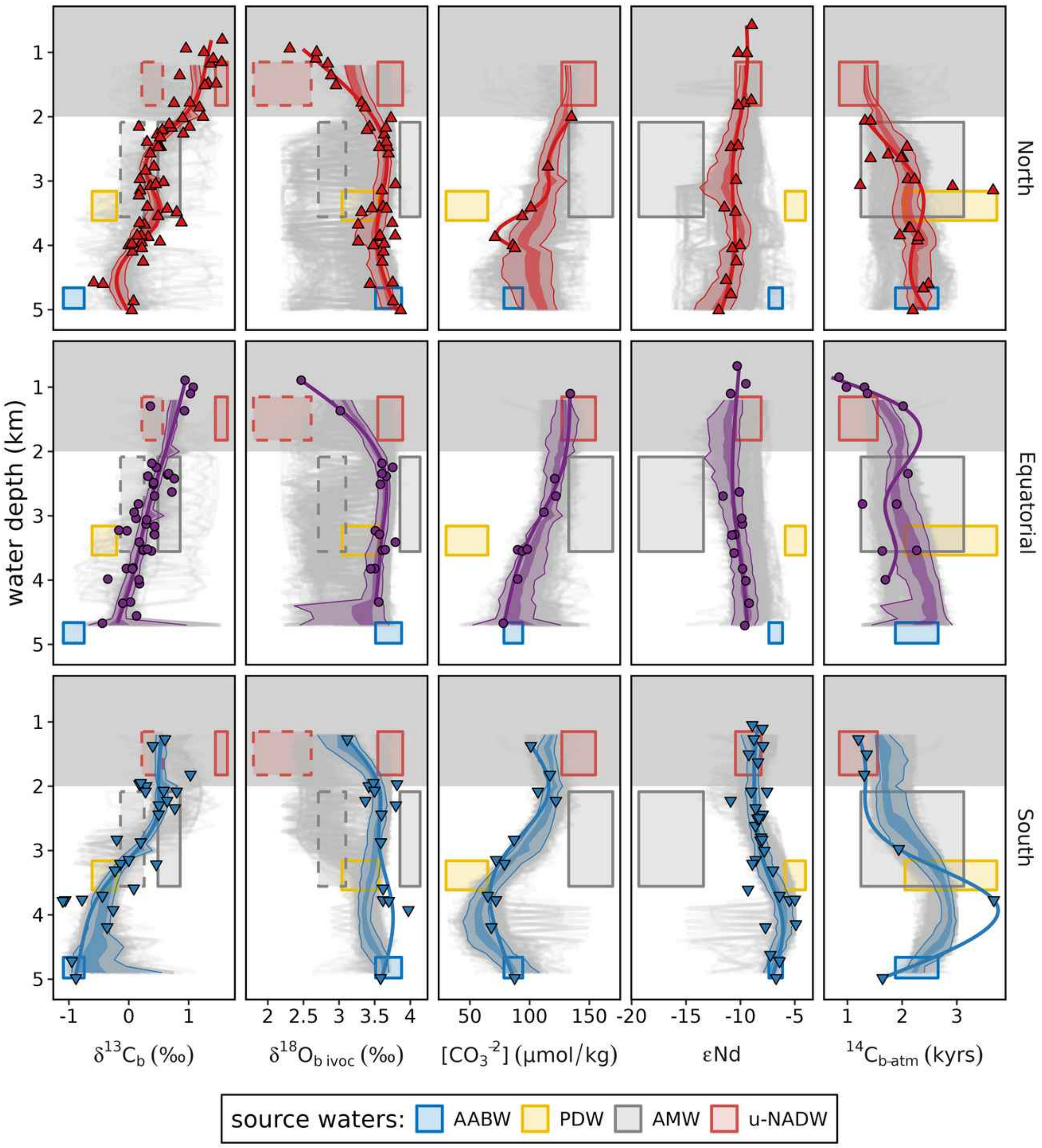

Supplementary Figure 9: *Same as Supplementary Fig. 7, but for the Last Glacial Maximum. Dashed rectangles indicate carbon and oxygen isotope signatures of mode 2 NADW source waters.*

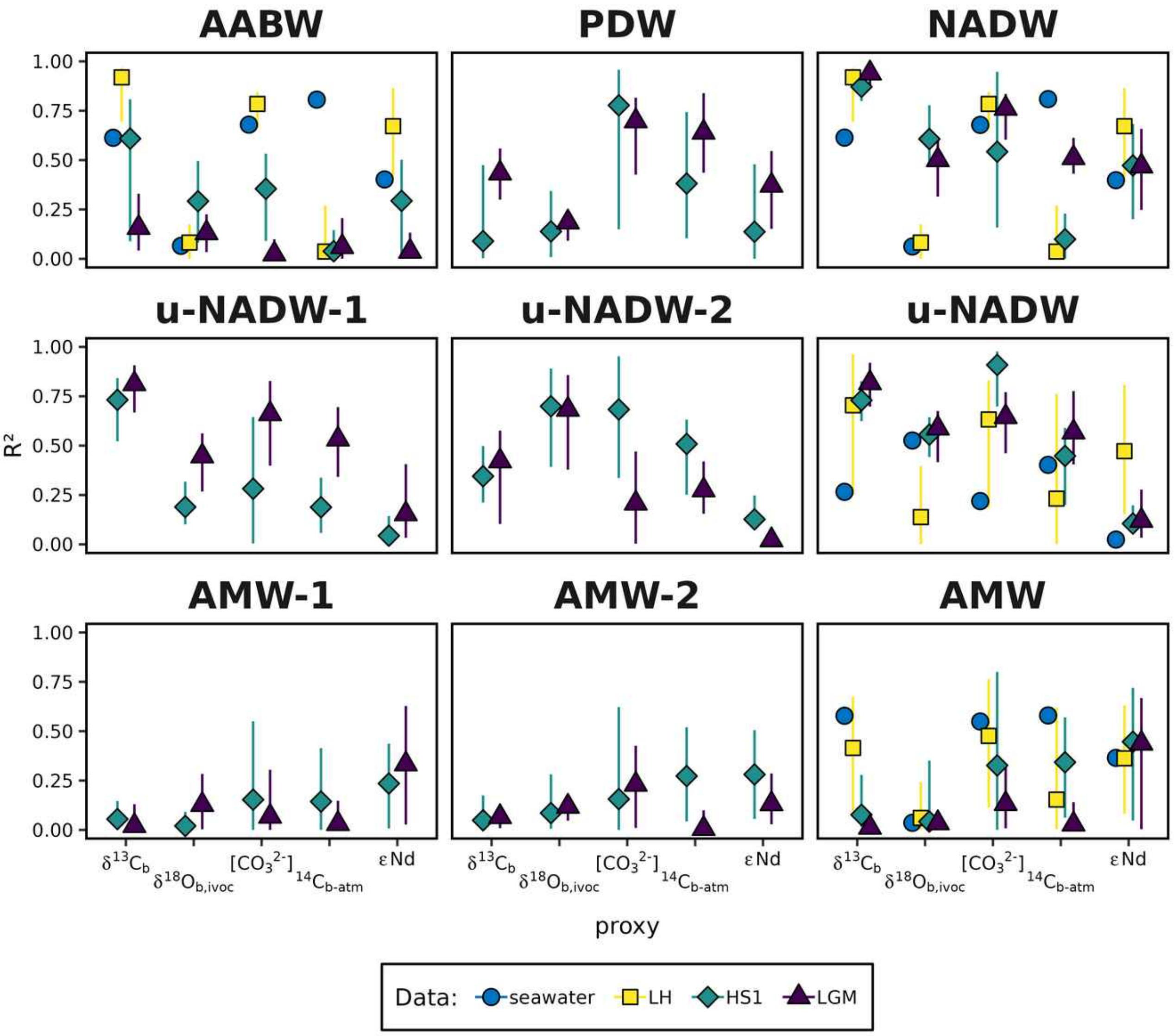

Supplementary Figure 10: *Performance of individual proxies to reconstruct source water contributions. Results for LH, HS1, and LGM are based on the depth-smoothed proxy data. The performance is expressed as linear correlation coefficient R² between observations from each individual proxy and estimated source water abundances. Symbols and error bars indicate mean and 95% uncertainty range of the best 20 % runs in the model ensemble. For seawater data, source water abundances are from* [25]*. R² here depends on several factors, including how distinct a source water proxy signature is and the distribution and quality of available observations.*

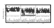

Supplementary Figure 11: *NADW contributions for the deep Atlantic as in Extended Data Fig. 9 during LH, HS1, and LGM for different ensemble subsets. Numbers indicate the number of model runs included in each subset. Background thick horizontal line in LH indicates calculations based on optimised multi parameter analysis (de Carvalho Ferreira and Kerr, 2017) and in HS1 and LGM represents results from* (Pöppelmeier et al., 2023). *Small black bars are "classical" scenarios based exclusively on* $\delta^{13}C_b$ *as proxy and AABW and u-NADW-1 as the only source waters.*

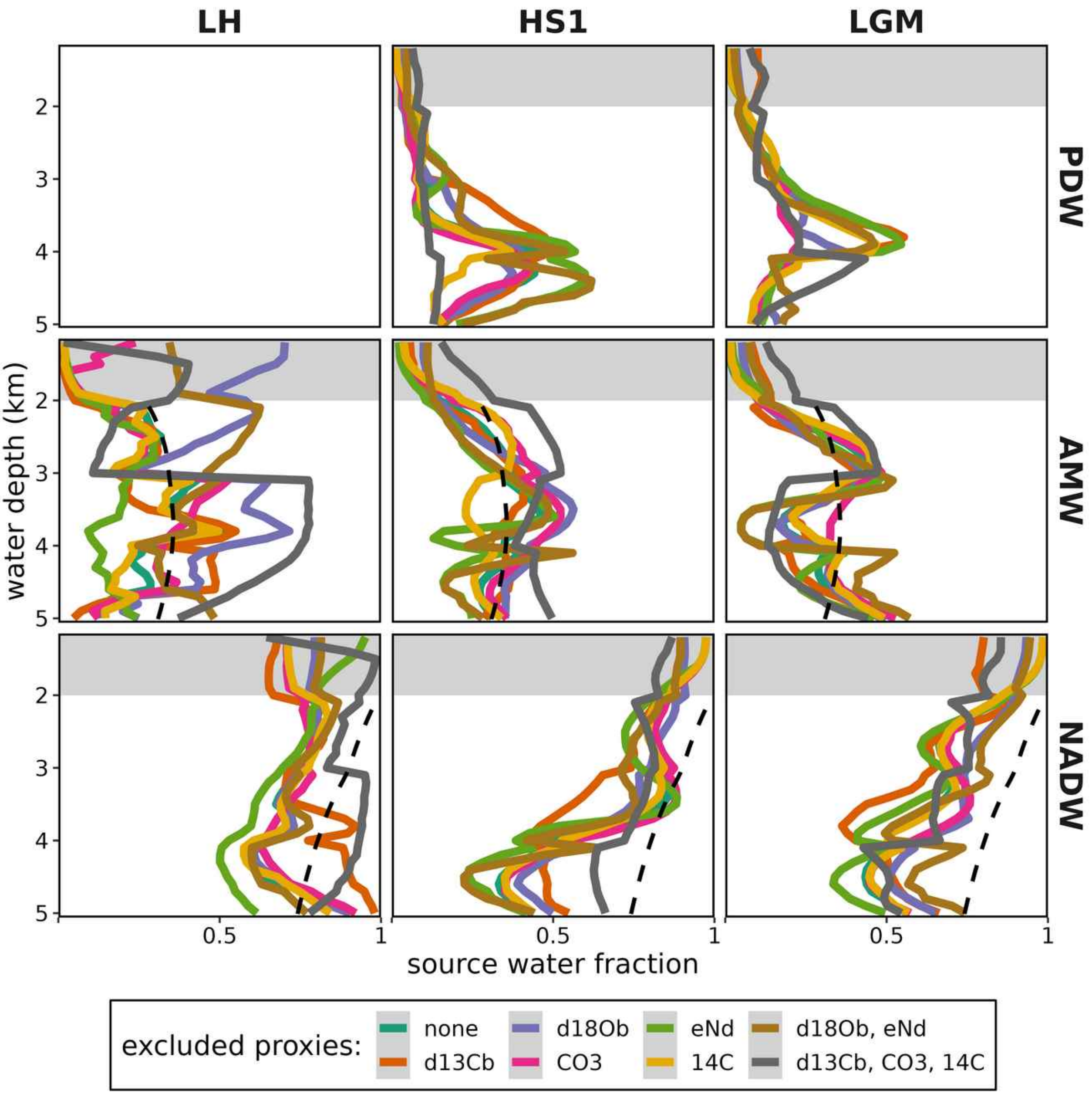

Supplementary Figure 12: *Source water fractions in the North Atlantic for different proxy combinations used, determined from the depth-smoothed data (Extended Data Fig. 6, similar to Fig. 3). Shown are vertical distributions of three key source waters in all time slices. Lines of different colours denote model sub-ensembles in which different proxies were excluded from all calculations. Black dashed lines denote the modern source water fractions, corrected for entrainment of u-NADW into l-NADW like in Fig. 3. Estimates at < 2 km water depth are not quantitative due to the neglect of Antarctic Intermediate Water (see Fig. 3). Estimate uncertainties are not shown, but lie in average at 0.04 ± 0.03 (central 50 % of data).*

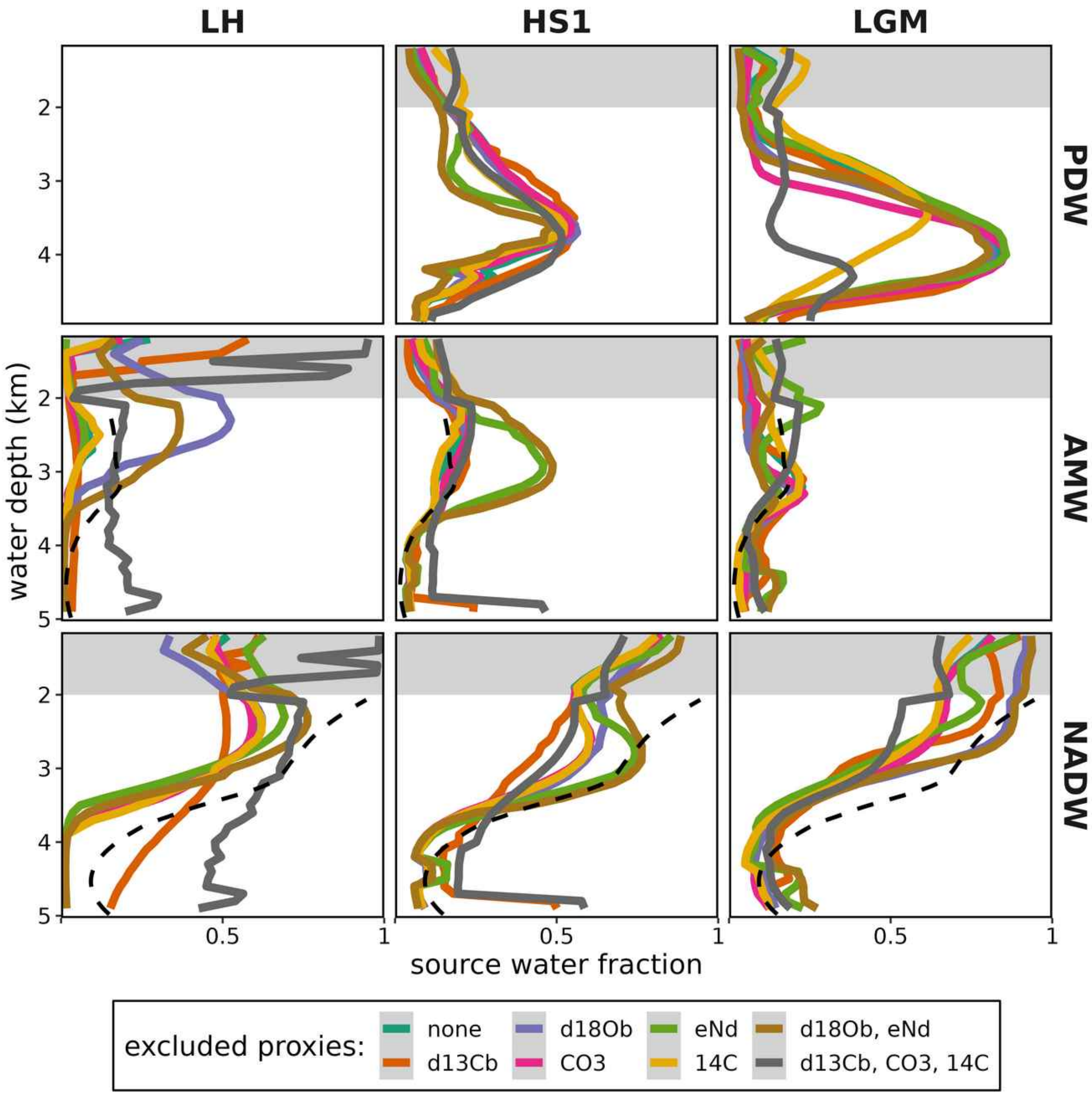

Supplementary Figure 13: *Same as Supplementary Fig. 12 but for the South Atlantic.*

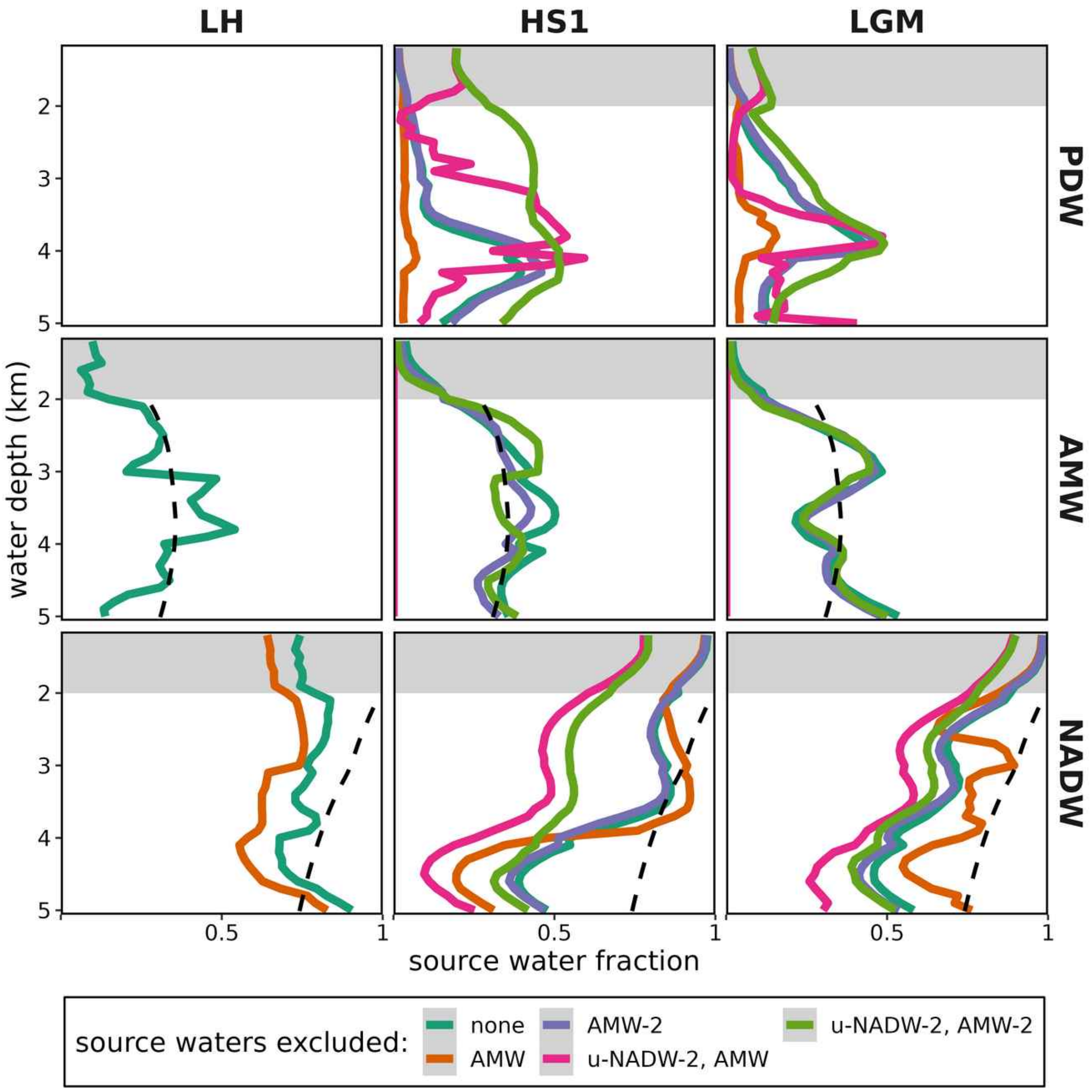

Supplementary Figure 14: *Same as Supplementary Fig. 12 but for different combinations of excluded source waters.*

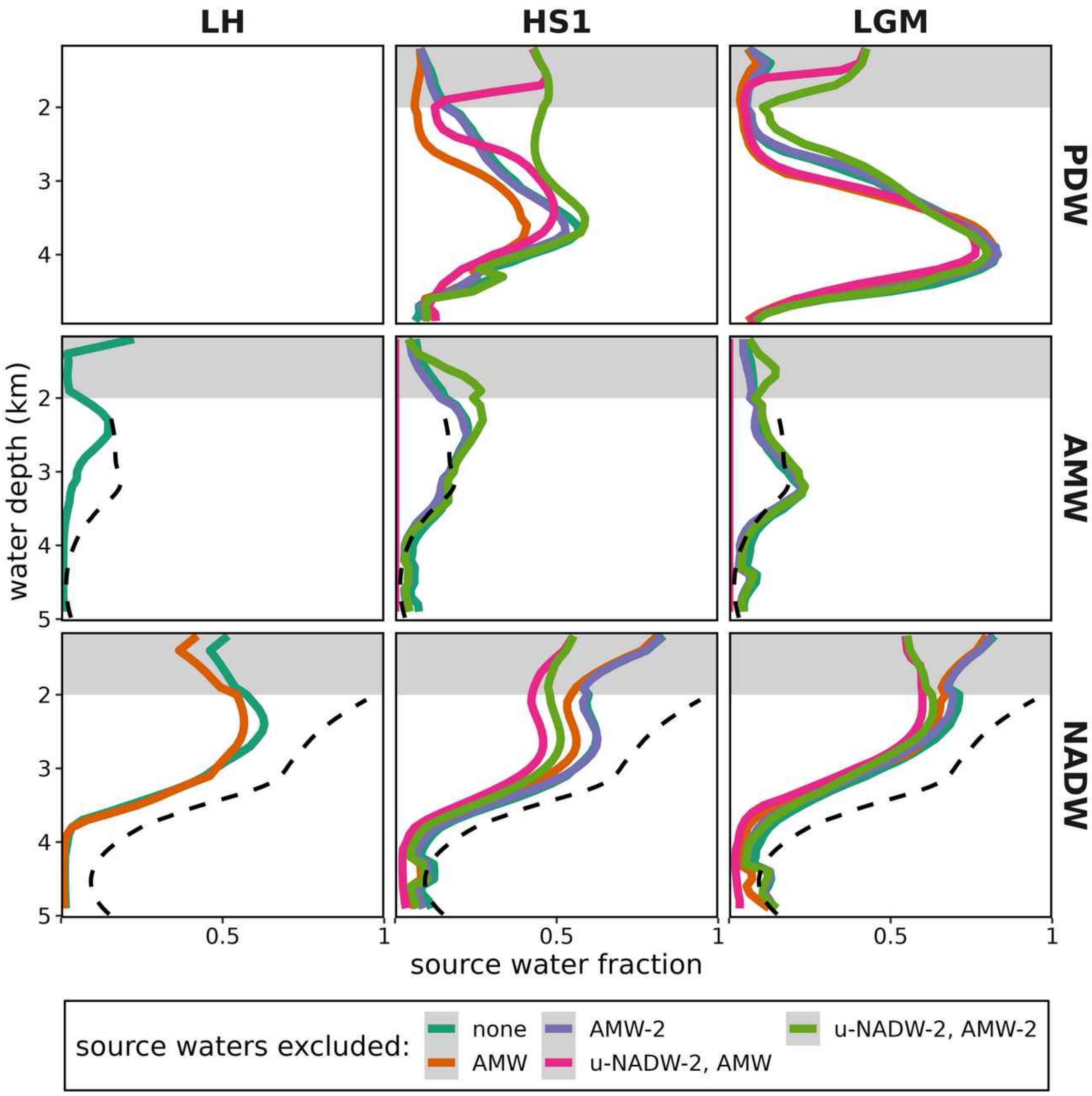

Supplementary Figure 15: *Same as Supplementary Fig. 12 but for different combinations of excluded source waters in the South Atlantic.*

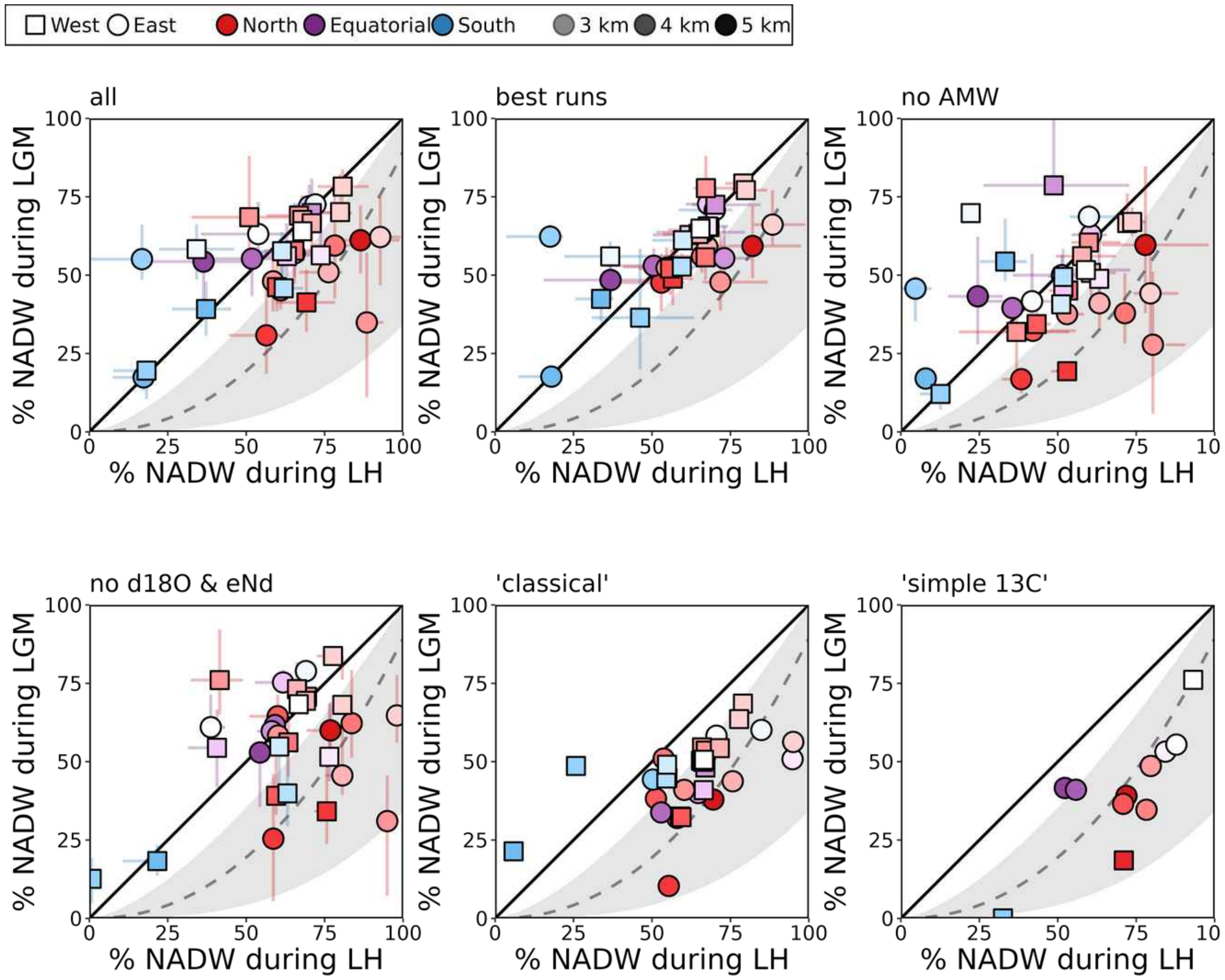

Supplementary Figure 16: *Comparisons of bulk NADW contributions in the box-pooled model during LGM and LH. Each panel shows a different subset of the simmr ensemble and two cases using only $\delta^{13}C_b$ as proxy and AABW and u-NADW-1 source waters ("classical" as simmr-based calculation and "simple 13C" as simple linear mixing calculation). "best runs" are the best fitting 20 % runs. Symbols, lines, and shading like in Extended Data Fig. 10.*

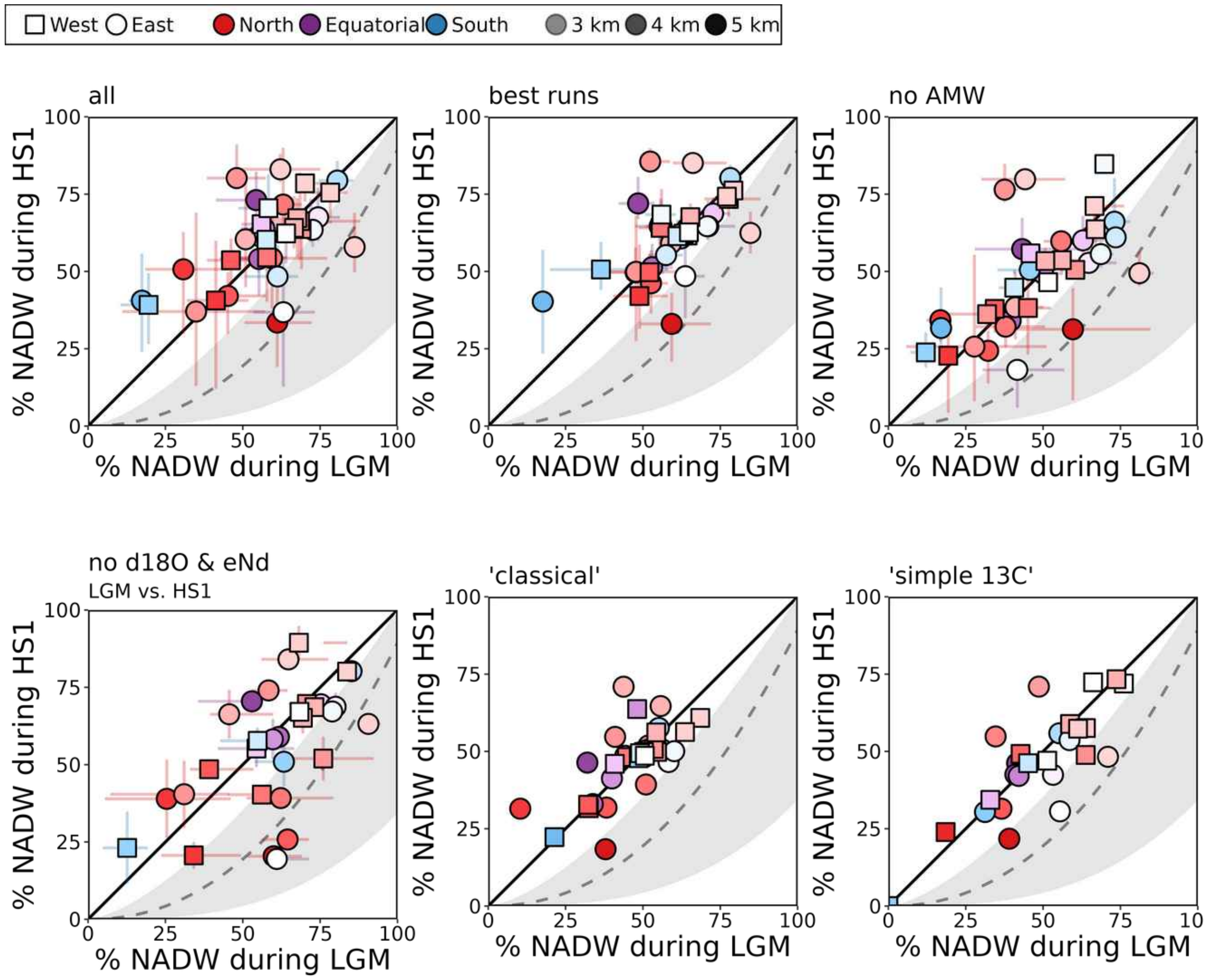

Supplementary Figure 17: *Comparisons of bulk NADW contributions in the box-pooled model during HS1 and LGM. Each panel shows a different subset of the simmr ensemble and two cases using only $\delta^{13}C_b$ as proxy and AABW and u-NADW-1 source waters ("classical" as simmr-based calculation and "simple 13C" as simple linear mixing calculation). "best runs" are the best fit runs. Symbols, lines, and shading like in Extended Data Fig. 10.*

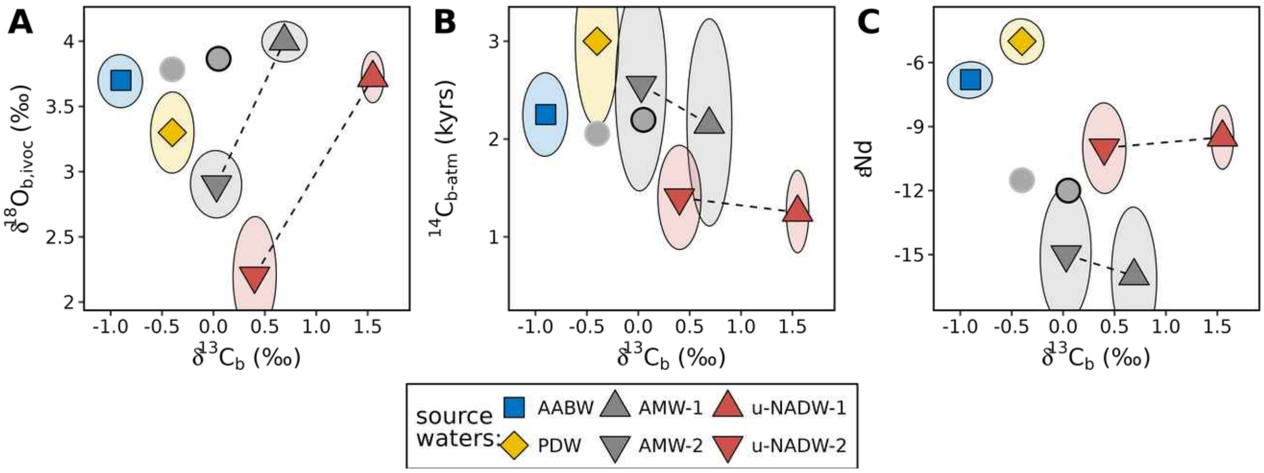

Supplementary Figure 18: *Mixing at abyssal north-west Atlantic Site KNR197-10-17. Same as Figure 2 but without [CO_3^{2-}] (because no data exist for the site) and with data for this site shown as grey circles with black and grey border for LGM and HS1, respectively. Coordinates for KNR197-10-17 are: 48.54°W, 36.405°N, 5010 m water depth* [29,30].

References used in this document

1. Duplessy, J.-C. *et al.* 13C Record of benthic foraminifera in the last interglacial ocean: Implications for the carbon cycle and the global deep water circulation. *Quaternary Research* **21**, 225–243 (1984).
2. Schmittner, A. *et al.* Calibration of the carbon isotope composition ($\delta^{13}C$) of benthic foraminifera. *Paleoceanography* **32**, 512–530 (2017).
3. Boyle, E. A. Cadmium: Chemical tracer of deepwater paleoceanography. *Paleoceanography* **3**, 471–489 (1988).
4. Oppo, D. W. *et al.* Data Constraints on Glacial Atlantic Water Mass Geometry and Properties. *Paleoceanography and Paleoclimatology* **33**, 1013–1034 (2018).
5. Gottschalk, J. *et al.* Carbon isotope offsets between benthic foraminifer species of the genus Cibicides (Cibicidoides) in the glacial sub-Antarctic Atlantic. *Paleoceanography* **31**, 2016PA003029 (2016).
6. Mackensen, A., Hubberten, H.-W., Bickert, T., Fischer, G. & Fütterer, D. K. The δ13C in benthic foraminiferal tests of Fontbotia wuellerstorfi (Schwager) Relative to the δ13C of dissolved inorganic carbon in Southern Ocean Deep Water: Implications for glacial ocean circulation models. *Paleoceanography* **8**, 587–610 (1993).
7. Yu, J. *et al.* Last glacial atmospheric CO 2 decline due to widespread Pacific deep-water expansion. *Nature Geoscience* 1–6 (2020) doi:10.1038/s41561-020-0610-5.
8. Rasmussen, T. L. & Thomsen, E. Stable isotope signals from brines in the Barents Sea: Implications for brine formation during the last glaciation. *Geology* **37**, 903–906 (2009).
9. Marchitto, T. M. *et al.* Improved oxygen isotope temperature calibrations for cosmopolitan benthic foraminifera. *Geochimica et Cosmochimica Acta* **130**, 1–11 (2014).
10. Shackleton, N. J. Attainment of isotopic equilibrium between ocean water and benthonic foraminifera genus Uvigerina : isotopic changes in the ocean during the last glacial. in *Les Méthodes Quantitatives D'Etude Des Variations Du Climat Au Cours Du Pléistocène* vol. 219 203 (Gif-sur-Yvette, 1974).
11. Dokken, T. M. & Jansen, E. Rapid changes in the mechanism of ocean convection during the last glacial period. *Nature* **401**, 458–461 (1999).

12. Ostermann, D. R. & Curry, W. B. Calibration of stable isotopic data: An enriched δ18O standard used for source gas mixing detection and correction. *Paleoceanography* **15**, 353–360 (2000).
13. Yu, J. & Elderfield, H. Benthic foraminiferal B/Ca ratios reflect deep water carbonate saturation state. *Earth and Planetary Science Letters* **258**, 73–86 (2007).
14. Yu, J., Elderfield, H. & Piotrowski, A. M. Seawater carbonate ion-δ13C systematics and application to glacial–interglacial North Atlantic ocean circulation. *Earth and Planetary Science Letters* **271**, 209–220 (2008).
15. Broecker, W. S. & Peng, T.-H. Gas exchange rates between air and sea. *Tellus* **26**, 21–35 (1974).
16. Skinner, L. C. & Bard, E. Radiocarbon as a Dating Tool and Tracer in Paleoceanography. *Reviews of Geophysics* **60**, e2020RG000720 (2022).
17. Key, R. M. *et al.* A global ocean carbon climatology: Results from Global Data Analysis Project (GLODAP). *Global Biogeochemical Cycles* **18**, (2004).
18. Khatiwala, S., Primeau, F. & Holzer, M. Ventilation of the deep ocean constrained with tracer observations and implications for radiocarbon estimates of ideal mean age. *Earth and Planetary Science Letters* **325–326**, 116–125 (2012).
19. Tachikawa, K. *et al.* The large-scale evolution of neodymium isotopic composition in the global modern and Holocene ocean revealed from seawater and archive data. *Chemical Geology* **457**, 131–148 (2017).
20. Blaser, P. *et al.* The resilience and sensitivity of Northeast Atlantic deep water εNd to overprinting by detrital fluxes over the past 30,000 years. *Geochimica et Cosmochimica Acta* **245**, 79–97 (2019).
21. Pöppelmeier, F. *et al.* Neodymium isotopes as a paleo-water mass tracer: A model-data reassessment. *Quaternary Science Reviews* **279**, 107404 (2022).
22. Blaser, P. *et al.* Labrador Sea bottom water provenance and REE exchange during the past 35,000 years. *Earth and Planetary Science Letters* **542**, 116299 (2020).
23. Pöppelmeier, F. *et al.* Influence of Ocean Circulation and Benthic Exchange on Deep Northwest Atlantic Nd Isotope Records During the Past 30,000 Years. *Geochemistry, Geophysics, Geosystems* **20**, 4457–4469 (2019).
24. Dickson, R. R. & Brown, J. The production of North Atlantic Deep Water: Sources, rates, and pathways. *Journal of Geophysical Research: Oceans* **99**, 12319–12341 (1994).

25. de Carvalho Ferreira, M. L. & Kerr, R. Source water distribution and quantification of North Atlantic Deep Water and Antarctic Bottom Water in the Atlantic Ocean. *Progress in Oceanography* **153**, 66–83 (2017).
26. Meland, M. Y., Dokken, T. M., Jansen, E. & Hevrøy, K. Water mass properties and exchange between the Nordic seas and the northern North Atlantic during the period 23-6 ka: Benthic oxygen isotopic evidence. *Paleoceanography* **23**, (2008).
27. Seidenkrantz, M.-S. *et al.* Evidence for influx of Atlantic water masses to the Labrador Sea during the Last Glacial Maximum. *Scientific Reports* **11**, 6788 (2021).
28. Yu, J. *et al.* Last glacial atmospheric CO2 decline due to widespread Pacific deep-water expansion. *Nat. Geosci.* **13**, 628–633 (2020).
29. Keigwin, L. D. & Swift, S. A. Carbon isotope evidence for a northern source of deep water in the glacial western North Atlantic. *Proceedings of the National Academy of Sciences* **114**, 2831–2835 (2017).
30. Pöppelmeier, F., Gutjahr, M., Blaser, P., Keigwin, L. D. & Lippold, J. Origin of Abyssal NW Atlantic Water Masses Since the Last Glacial Maximum. *Paleoceanography and Paleoclimatology* **33**, 530–543 (2018).
31. Lambeck, K., Rouby, H., Purcell, A., Sun, Y. & Sambridge, M. Sea level and global ice volumes from the Last Glacial Maximum to the Holocene. *PNAS* **111**, 15296–15303 (2014).
